# Disruptive natural selection by male reproductive potential prevents underexpression of protein-coding genes on the human Y chromosome as a self-domestication syndrome

**DOI:** 10.1186/s12863-020-00896-6

**Published:** 2020-10-22

**Authors:** Mikhail Ponomarenko, Maxim Kleshchev, Petr Ponomarenko, Irina Chadaeva, Ekaterina Sharypova, Dmitry Rasskazov, Semyon Kolmykov, Irina Drachkova, Gennady Vasiliev, Natalia Gutorova, Elena Ignatieva, Ludmila Savinkova, Anton Bogomolov, Ludmila Osadchuk, Alexandr Osadchuk, Dmitry Oshchepkov

**Affiliations:** 1 Institute of Cytology and Genetics, Siberian Branch of Russian Academy of Sciences, 10 Lavrentyev Ave, Novosibirsk, 630090 Russia; 2 Novosibirsk State University, 1, Pirogova str., Novosibirsk, 630090 Russia

**Keywords:** Reproductive potential, Human, Y chromosome, Gene, Promoter, TATA box, TATA-binding protein, Single-nucleotide polymorphism, Candidate SNP marker, Verification

## Abstract

**Background:**

In population ecology, the concept of reproductive potential denotes the most vital indicator of chances to produce and sustain a healthy descendant until his/her reproductive maturity under the best conditions. This concept links quality of life and longevity of an individual with disease susceptibilities encoded by his/her genome. Female reproductive potential has been investigated deeply, widely, and comprehensively in the past, but the male one has not received an equal amount of attention. Therefore, here we focused on the human Y chromosome and found candidate single-nucleotide polymorphism (SNP) markers of male reproductive potential.

**Results:**

Examining in silico (i.e., using our earlier created Web-service SNP_TATA_Z-tester) all 1206 unannotated SNPs within 70 bp proximal promoters of all 63 Y-linked genes, we found 261 possible male-reproductive-potential SNP markers that can significantly alter the binding affinity of TATA-binding protein (TBP) for these promoters. Among them, there are candidate SNP markers of spermatogenesis disorders (e.g., rs1402972626), pediatric cancer (e.g., rs1483581212) as well as male anxiety damaging family relationships and mother’s and children’s health (e.g., rs187456378). First of all, we selectively verified in vitro both absolute and relative values of the analyzed TBP–promoter affinity, whose Pearson’s coefficients of correlation between predicted and measured values were r = 0.84 (significance *p* <  0.025) and r = 0.98 (p <  0.025), respectively. Next, we found that there are twofold fewer candidate SNP markers decreasing TBP–promoter affinity relative to those increasing it, whereas in the genome-wide norm, SNP-induced damage to TBP–promoter complexes is fourfold more frequent than SNP-induced improvement (*p* <  0.05, binomial distribution). This means natural selection against underexpression of these genes. Meanwhile, the numbers of candidate SNP markers of an increase and decrease in male reproductive potential were indistinguishably equal to each other (*p* <  0.05) as if male self-domestication could have happened, with its experimentally known disruptive natural selection. Because there is still not enough scientific evidence that this could have happened, we discuss the human diseases associated with candidate SNP markers of male reproductive potential that may correspond to domestication-related disorders in pets.

**Conclusions:**

Overall, our findings seem to support a self-domestication syndrome with disruptive natural selection by male reproductive potential preventing Y-linked underexpression of a protein.

## Background

In keeping with Royal Chapman’s [1] and Eric Pianka’s [2] ideas, now populational ecologists use the concept of reproductive potential as the most vital indicator of the best-condition chances to reproduce own descendant and sustain him/her until his/her reproductive maturity in the next generation at individual and population levels [3]. Bowles’ theory of life expectancy [4] links the reproductive potential, quality of life, and longevity of an individual with resistance to diseases and stressors as encoded by his/her genome. Thus, progress of medicine, advances in sciences, technology developments, and better education can increase whereas an increase in environmental pollution, the growth of urbanization, growing population, infection and parasite epidemics can decrease the reproductive potential of an individual.

To find out how a person can increase one’s own and offspring’s quality of life and longevity, predictive-preventive personalized participatory medicine [5] uses the fundamental concept of clinical single-nucleotide polymorphism (SNP) markers, which significantly differ between the cohorts of patients and conventionally healthy volunteers (see, e.g. [6]). A physician uses SNP markers of reproductive potential in individual genomes of his/her patients to tell them about diseases and stress factors that can worsen their health, longevity, and quality of life and those of their offspring as well as what kinds of lifestyles, prevention/recovery actions, medications, diets, and physician–patient mutual help allow for avoiding these dangers.

The cornerstone of this postgenome medicine is the greatest twenty-first-century scientific project “1000 Genomes” [7], which has already identified many hundreds of millions of SNPs (i.e., database dbSNP [8]) as deviations of many thousands of known individual genomes [9] from their assembly in the reference human genome (i.e., database Ensembl [10]), which are all available to the public thanks to the UCSC Genome Browser [11]. Finally, database dbWGFP [12] compiles, systematizes, and prioritizes any data on each of the 10 billion potential genome-wide SNPs in humans that may help physicians to deal with individual genomes of their patients.

Because a physician’s decision based on a patient’s individual genome affects health, quality of life, and longevity of this patient, only those biomedical SNP markers are suitable for this decision that are clinically proved by a comparison between cohorts of diseased and healthy people. Considering how much time, manual labor, and funding is required, this task actually seems impossible because each of the 10 billion human SNPs [12] may manifest itself during pathogenesis of each of the 55,000 diseases listed in the 11th International Statistical Classification of Diseases and Related Health Problems (ICD-11) [13]. Nonetheless, it seems debatable whether it is necessary to test each human SNP clinically, given that the absolute majority of them do not affect health in any way, in line with Kimura’s theory of neutral evolution [14] and Haldane’s dilemma [15]. For future clinical verification, in relation to any given disease, the mainstream strategy doubtlessly is the supervised manual selection of a candidate SNP marker among all the unannotated SNPs near the human genes that are already associated with this disease [6]. Furthermore, a cohort-based clinical search for biomedical SNP markers may be much more rapid, low-cost, and focused if prior computations (genome-wide) can ignore the absolute majority of neutral SNPs among all the unannotated SNPs [16]. Indeed, in silico accuracy of annotation still seems to be quite modest for application to clinical practice [17] but increases every year (e.g., [18–23]).

As for accuracy of annotation, at present, the best one is achieved with SNPs in protein-coding regions of genes [24]; these SNPs damage proteins irreparably [25]. The worst accuracy of annotation is associated with regulatory SNPs [26], which modulate protein levels, which are correctable by lifestyle changes and medications. Therefore, regulatory SNPs in TBP-binding sites (TBP-sites) seem to be promising in terms of both biomedical usefulness and predictability [16, 27] owing to their obligatory presence upstream of any transcription start site [28, 29], and these SNPs increase gene expression proportionally with the TBP–promoter affinity altered by them [16, 30]. The canonical form of a TBP-site, TATA box, represents ~ 15% of TBP-sites and is the best-studied regulatory genomic site in eukaryotes [27].

Previously, we have created Web service SNP_TATA_Comparator [31] and used it to predict a number of candidate SNP markers in TBP-sites in relation to obesity [32], aggressiveness [33], chronopathology [34], atherosclerosis [35], resistance to anticancer treatment [36], autoimmune diseases [37], Alzheimer’s disease [38], and social domination/subordination [39] in humans. In order to extend the areas of its application, here our aim was to find candidate SNP markers of male reproductive potential on the human Y chromosome and to compare their prevalence rates with the commonly accepted genome-wide norm because female reproductive potential has been thoroughly studied earlier (including our recent work [40]), but the male one has not received an equal amount of attention yet.

## Results and discussion

Using our public Web service SNP_TATA_Z-tester [41], we analyzed all 1206 SNPs of the 70 bp proximal promoters of all the 63 protein-coding genes on the human Y chromosome that are publicly available in the human reference genome GRCh38 [10] and dbSNP, rel. 151 [8]. As a result, we predicted 261 candidate SNP markers of male reproductive potential among the 1206 SNPs under study (Table 1). Tables S1–S4 (hereinafter: see Supplementary Results, Additional file 1) show these predictions. As one can see in Tables 1, 31 of the 63 genes analyzed (i.e., *BPY2*, *BPY2B*, *BPY2C*, *CDY1*, *CDY1B*, *CDY2B*, *DAZ1*, *DAZ2*, *DAZ3*, *DAZ4*, *DDX3Y*, *EIF1AY*, *HSFY1*, *HSFY2*, *PCDH11Y*, *PRKY*, *PRY*, *PRY2*, *RBMY1A1*, *RBMY1B*, *RBMY1D*, *RBMY1E*, *RBMY1F*, *RBMY1J*, *RPS4Y1*, *SRY*, *TGIF2LY*, *TSPY1*, *TSPY3*, *TSPY9P*, and *VCY1B*) contain 75 unannotated SNPs that were studied here, but none of them was not predicted as a candidate SNP marker (data not shown).

**Table 1:** Candidate SNP markers of male reproductive potential in the human Y-linked protein-coding genes and their comparison with the genome-wide patterns

Data: GRCh38, dbSNP rel. 151 [8]			Result	H_0_: neutral natural selection			H_0_: ↑♂ and ↓♂ sameness		
Human body systems	N_GENE_	N_SNP_	N_RES_	N_>_	N_<_	*P*(N_<_≡4 N_>_ ≡ 4N_RES_/5)	N_↑_	N_↓_	*P*(N_↑_ ≡ N_↓_ ≡ N_RES_/2)
Whole-genome norm for SNPs within TF-sites [42]	10^4^	10^5^	1000	200	800	> 0.52			
Clinical SNP markers for diseases in TBP-sites [31]	33	203	51	14	37	> 0.93			
Candidate SNP markers mainly for female reproductive potential in TBP-sites [40]	22	129	24	19	5	< 0.000001			
Y-linked genes^@^ in PAR1 (pseudo-autosomal region 1)	15	899	211	143	68	< 0.000001	101	110	> 0.2
Y-linked genes in PAR2 (pseudo-autosomal region 2)	3	135	25	20	5	< 0.000001	10	15	> 0.1
Male-specific Y-linked genes^@^ paralogous to the appropriate X-linked genes	8	56	13	8	5	< 0.01	4	9	> 0.1
Male-specific Y-linked unique genes^@^	6	41	12	6	6	< 0.025	4	8	> 0.1
Y-linked protein-coding genes^@^	32	1131	261	176	85	< 0.000001	119	142	> 0.06
Other Y-linked protein-coding genes in humans	31	75	–	–	–		–	–	–
**TOTAL**	**63**	**1206**	**261**	176	85	< 0.000001	119	142	> 0.06

**Notes:** ♂, male reproductive potential: increased (↑) and reduced (↓); N_GENE_ and N_SNP_, total numbers of the human genes and of their SNPs meeting the criteria of this study. N_RES_, the total number of the candidate SNP markers predicted in this work that can increase (N_>_) or decrease (N_<_) the affinity of TATA-binding protein (TBP) for these promoters and hence the expression of these genes. N_↑_ and N_↓_, the total numbers of the candidate SNP markers that can increase or decrease male reproductive potential, respectively. *P*(H_0_), the estimate of probability for the acceptance of this H_0_ hypothesis, for a binomial distribution; TF-site, transcription factor–binding site; ^@^genes whose expression can be significantly altered by SNPs of their TBP-sites

### Pseudo-autosomal region 1 (PAR1) of the human Y chromosome

**Fig. 1:**
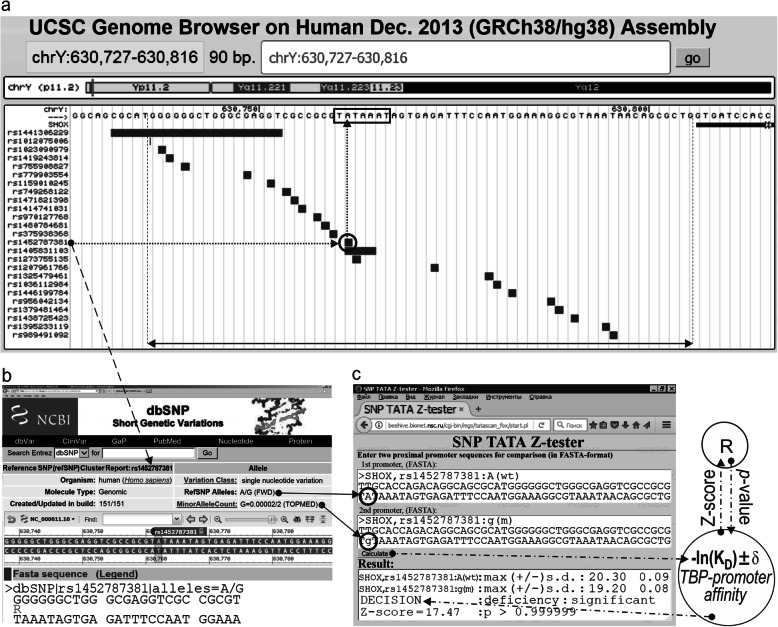
The result calculated by SNP_TATA_Z-Tester [41] for candidate SNP marker rs1452787381 of male reproductive potential within the human *SHOX* gene. **a** Unannotated SNPs (analyzed in this study) in the 70 bp proximal promoter (where all proven TBP-sites [boxed] are located; double-headed arrow, ↔) of the human *SHOX* gene retrieved from dbSNP, rel. 151 [8], using the UCSC Genome Browser [11]. Dotted arrow: unannotated SNP rs1452787381 retrieved from dbSNP (**b**) can be a candidate SNP marker of male reproductive potential as we are predicting here because of a significant change in the affinity of TBP for the human *SHOX* gene promoter (**c**). Solid arrows: data input into the two textboxes of our Web service SNP_TATA_Z-tester [41] as two DNA sequence variants of the ancestral (norm, wt) and minor (m) alleles of the SNP under study. Dash-and-dot arrows: estimates of significance of the change in gene product abundance in patients carrying the minor allele (relative to the norm), expressed as a Z-score using the R software [43]. Circles indicate the ancestral and minor alleles of the candidate SNP marker under study

**The human** ***SHOX*** **gene** encodes short stature homeobox (transcription factor). Figure 1 shows how we predicted candidate SNP markers for male reproductive potential within 70 bp proximal promoters (a double-headed arrow, ↔) of this gene, as detailed within instruction manuals [31, 41] of our Web-service. Here, line “Decision” of the “Results” textbox carries the label “deficiency: significant,” which is our prediction in the case of unannotated SNP rs1452787381 (Fig. 1c). This text means that the minor allele of the SNP under study (rs1452787381) decreases TBP–promoter affinity in comparison with the wild-type ancestral allele, which can manifest itself as underexpression of the *SHOX* gene containing the above-mentioned minor allele.

Table S1 (see Supplementary Results, Additional file 1) documents this prediction in columns entitled “K_D_, nM,” namely: *K*_*D*_ values of the equilibrium dissociation constant of complexes of TBP and one of the two entered alleles of the promoter under study and their standard errors in nanomoles per liter (nM). Additionally, this table shows a change (Δ) in gene expression and its Fisher’s *Z*-score with statistical significance α, as described elsewhere (see Supplementary Methods, Additional file 2). Finally, there is heuristic prioritization rank ρ displayed in alphabetical order from the “best” (A) to the “worst” (E).

**Fig. 2:**
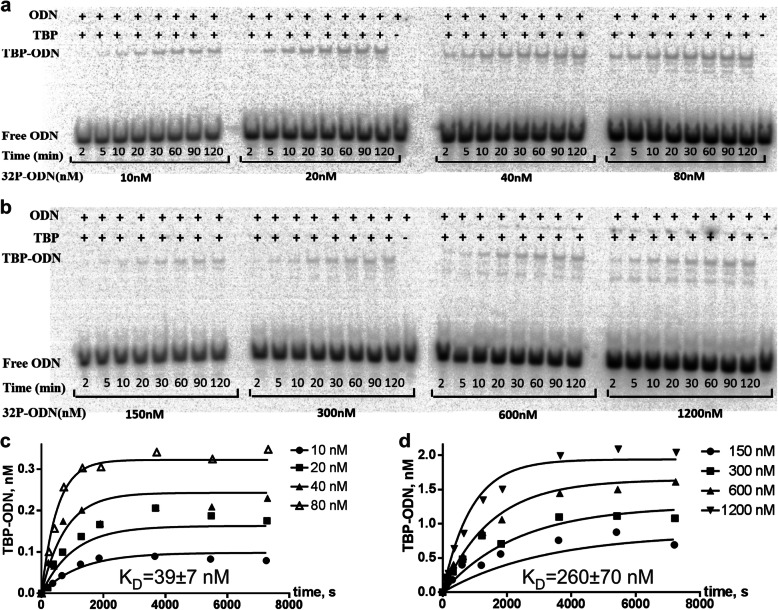
Measuring the kinetics of TBP binding to two TATA-containing ODNs identical to the human *SHOX* gene promoter. **a** and **b** Electropherograms in the cases of the wild-typed ancestral and minor alleles of the unannotated SNP rs1452787381 under this study, respectively; the concentration of TBP was 2 nM in all the experiments; the concentrations of an ODN containing a tested SNP allele that we used are indicated; **c** and **d** dependences of reaction rates on ODN concentrations in the cases of the ancestral and minor alleles of the SNP rs1452787381, respectively; the *K*_D_ value of the equilibrium dissociation constant was inferred from the dependences of reaction rates on ODN concentrations according to publicly available software GraphPad Prism 5 (http://graphpad-prism.software.informer.com/5.01)

First, we verified this prediction in vitro using an electrophoretic mobility shift assay (EMSA) as described within the subsection “In vitro verification” of the main section “Methods.” Figure 2 presents the result of this verification (also, see Additional file 4: Supplementary Electropherograms).

As shown in this figure, TBP–DNA affinity decreased from 39 ± 7 nM for the synthetic oligonucleotide (ODN) identical to the wild-type allele of SNP rs1452787381 being tested (Fig. 2c) to 260 ± 70 nM in the case of the minor allele of this SNP (Fig. 2d), as predicted in silico (Fig. 1c). This means that our Web service [41] is applicable to studies on genes on the human Y chromosome.

In the order of discussion, three rightmost columns of Table S1 cite a clinical report [44] on *SHOX* deficiency as a known physiological marker of Madelung deformity and disproportionate short stature in newborns, as found in the PubMed database in its “Keyword search” mode [45] (hereinafter: see Supplementary Keyword Search, Additional file 3). That is why there is a down arrow (↓) in the “♂” column, which means a candidate SNP marker for a decrease in male reproductive potential, as predicted here, within the applicability limits described in ref. [44] without any heuristic assumptions.

In total, we thus found five SNPs decreasing *SHOX* expression as candidate SNP markers of a decrease in male reproductive potential in accordance with ref. [44] (Table S1: e.g., rs771395540). Similarly, we revealed three SNPs causing *SHOX* overexpression, which is a clinical physiological marker of pathoembryogenesis according to another clinical report found [46]. In this way, we predicted three candidate SNP markers decreasing male reproductive potential too, as shown in Table S1 (e.g., rs28378830).

**The human** ***ZBED1*** **gene** encodes zinc finger BED-type domain–containing protein and contains a single SNP (rs1358454071) that corresponds to *ZBED1* underexpression, whereas 11 SNPs (e.g., rs1317376848) cause its overexpression, as calculated here (Table S1). By searching PubMed, we found clinical data [47] on the dual role of *ZBED1* in the adenovirus life cycle, namely, its overexpression and underexpression promote infection of uninfected spermatozoa and virus overproduction during late stages of the viral life cycle, respectively, whereas adenovirus infection of spermatozoa is a risk factor for male infertility and spontaneous abortion [48] (Table 2). Using these two clinical findings [47, 48] taken together, we predicted 12 candidate SNP markers of a loss of male reproductive potential, as presented in Table S1.

**Table 2:** EMSA-based in vitro analysis of a complex of TBP and one of synthetic 26 bp ODNs identical to natural promoters near the SNPs being tested

Gene, dbSNP ID [8]	Allele:WT *min*	26 bp oligodeoxyribonucleotides (ODNs), 5′ → 3′	Prediction		Experiment		
			-ln (K_D_) ln-unit	Δln (K_D_) ln-unit	K_D_ ± SEMnM	-ln (K_D_) ln-unit	Δln (K_D_) ln-unit
*SHOX*	-45A	gaggtcgccgcgt**A**taaatagtgaga	20.31	−1.10	39 ± 7	17.06	−1.90
rs1452787381	-*45G*	gaggtcgccgcgt**G**taaatagtgaga	19.21		260 ± 70	15.16	
*GTPBP6*	-24G	atcacgagcacgt**G**atgaggagcggc	17.30	1.38	1500 ± 200	13.41	0.07
rs1393008234	*-24 T*	atcacgagcacgt**T**atgaggagcggc	18.68		1400 ± 200	13.48	
*ASMT*	-30G	ggtgaccttttgt**G**cccagaataggt	18.18	0.75	600 ± 300	14.33	−0.51
rs1402972626	*-30A*	ggtgaccttttgt**A**cccagaataggt	18.93		1000 ± 300	13.82	
*ZFY*	-56C	ggcggagggggcc**C**aactaccatccc	17.67	0.51	1000 ± 400	13.82	−0.70
rs1452787381	*-56 T*	ggcggagggggcc**T**aactaccatccc	18.18		2000 ± 1000	13.12	
*CDY2A*	-24G	agaatgttccata**T**aatcgtcatagc	19.27	−0.51	160 ± 30	15.65	−1.14
rs20067072	*-24 t*	agaatgttccata**C**aatcgtcatagc	18.76		500 ± 200	14.51	

**Notes.** For each TBP–ODN complex: K_D_, equilibrium dissociation constant; SEM, standard error of the mean. All experimental data and their SEMs are the output of publicly available software GraphPad Prism 5 (URL = http://graphpad-prism.software.informer.com/5.01), the input data of which were the dependences of reaction rates on ODN concentrations, as illustrated in Fig. 2c and d

**The human** ***AKAP17A*** **gene** (A-kinase anchoring protein 17A) contains 13 SNPs (e.g., rs1420856028) that can elevate the expression of this gene, as shown in Table S1. For *AKAP17A* overexpression, our PubMed keyword search retrieved transcriptome data on azoospermia caused by testicular degeneration in Klinefelter syndrome [49], where AKAP17A overexpression is the best physiological marker of this pathology. Accordingly, we propose 13 candidate SNP markers of a decrease in male reproductive potential (Table S1). In addition, we found six SNPs (e.g., rs1397856076:c,) causing *AKAP17A* underexpression, which is protective against azoospermia in Klinefelter syndrome [49]. Thus, we predicted six candidate SNP markers of an increase in male reproductive potential, which are listed in Table S1.

**The human** ***P2RY8*** **gene** (P2Y receptor family member 8) contains two SNPs (rs1225019830 and rs1469023312) that cause its overexpression, while two others (rs1265835746 and rs1485298348) cause its underexpression, as predicted here (Table S1). After a PubMed keyword search, we learned that *P2RY8* overexpression is a physiological marker of iron excess in the human body [50]; this aberration reduces sperm quality via acceleration of oxidative DNA damage [51] and vice versa. Therefore, we propose that rs1225019830 and rs1469023312 are candidate SNP markers of a decrease in male reproductive potential and that rs1265835746 and rs1485298348 are candidate SNP markers of its increase (Table S1).

**Fig. 3:**
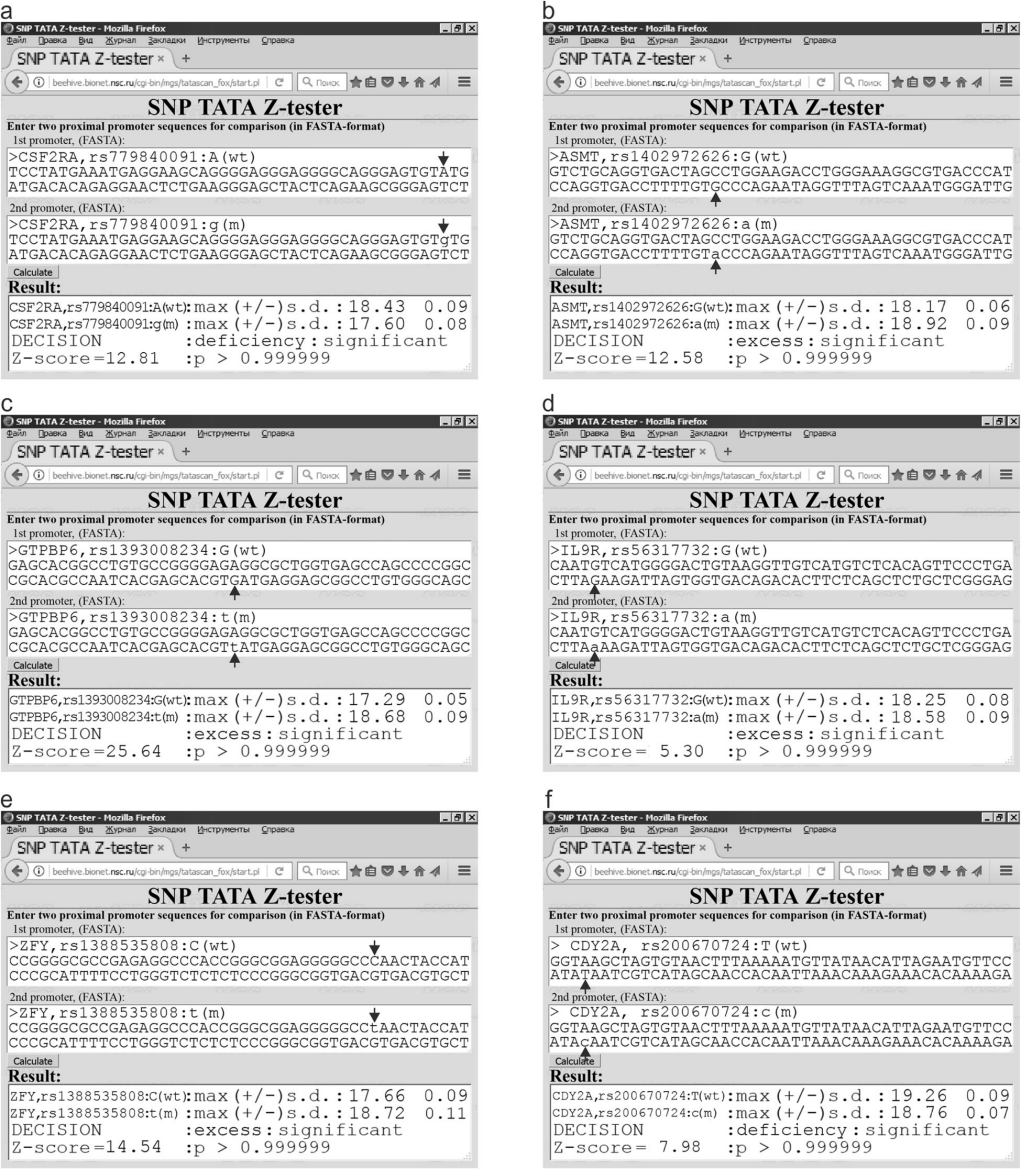
Examples of our predictions in this work in the case of human Y-linked genes. **a** The *CSF2RA* gene: rs779840091; **b** *ASMT*: rs1402972626; **c** *GTPBP6*: rs1393008234; **d** *IL9R:* rs56317732; **e** *ZFY*: rs1388535808; and **f** *CDY2A*: rs200670724

**Human genes** ***CSF2RA*,** ***CRLF2*, and** ***IL3RA*** respectively encode colony-stimulating factor 2 receptor subunit α, cytokine receptor–like factor 2, and interleukin 3 receptor subunit α. Our PubMed keyword search yielded three clinical studies [52–54] that uncovered a higher risk of pediatric leukemia in the case of overexpression of these receptors and vice versa (Table S1). We found 15 SNPs reducing the expression of these genes (Table S1: e.g., rs779840091), as exemplified in Fig. 3a.

Using the above-mentioned articles [52–54], we predicted these SNPs to be candidate SNP markers of an increase in male reproductive potential (Table S1). In addition, we propose 11 SNPs (e.g., rs1233753904) that can cause overexpression of these receptors as candidate SNP markers of a decrease in male reproductive potential, in line with the articles found [52–54] (Table S1).

**The human** ***GTPBP6*** **gene** encoding GTP-binding protein 6 contains three SNPs (e.g., rs1393008234) that can elevate GTPBP6 expression, whereas three other SNPs (e.g., rs1336077354) can downregulate it according to our calculations, as summarized in Table S1 and illustrated in Fig. 3c. A PubMed keyword search revealed two negative correlations, namely, between the GTPBP6 level and verbal IQ [55] as well as between verbal IQ and numbers of offspring and siblings [56]. Through these correlations [55, 56], we predicted two trios of candidate SNP markers presented in Table S1 that increase and decrease male reproductive potential, respectively.

**The human** ***CD99*** **gene** (CD99 molecule, synonym: Xg blood group) contains 20 and three SNPs corresponding to over- and underexpression of this gene, respectively (Table S1). After a PubMed keyword search, we found the clinical data [57] on *CD99* overexpression (in males versus females) that can elevate the risk of death in men with septic shock. That is why we predicted three candidate SNP markers (e.g., rs916987392) of an increase in male reproductive potential as well as 20 candidate SNP markers (listed in Table S1) decreasing it.

**The human** ***SLC25A6*** **gene** codes for solute carrier family 25 member 6. According to a PubMed keyword search, *SLC25A6* underexpression inhibits apoptosis [58] as a spermatogenesis disorder of spermatocytes [59]. On the basis of the cited data together with the output of our Web service [41], we proposed four candidate SNP markers (e.g., rs1240336670) of a reduction in male reproductive potential and rs1278813527 increasing it, as presented in Table S1.

**The human** ***PLCXD1*** **gene** encodes phosphatidylinositol-specific phospholipase C X domain–containing 1 and has 35 and 15 SNPs that can respectively elevate and reduce the transcription of this gene, as shown in Table S1. Judging by Affymetrix data [60], *PLCXD1* underexpression is a physiological marker of health status in men 5 h after a stroke. This observation allows us to heuristically predict 50 candidate SNP markers of male reproductive potential, positive and negative effects of which are presented in Table S1.

**The human** ***ASMT*** **gene** codes for acetylserotonin O-methyltransferase and contains 10 and three SNPs corresponding to *ASMT* overexpression and underexpression, which are listed in Table S1, as predicted here and depicted in Fig. 3b. For this melatonin synthesis enzyme, a PubMed keyword search revealed that melatonin circadian excess in testes is a daytime protector against oxidative DNA damage during spermatogenesis [61]. This finding allows us to propose 10 candidate SNP markers (e.g., rs1402972626) of an increase in male reproductive potential (Table S1). By the same reasoning [61], rs1313192261, rs1280760292, and rs1270130345 listed in Table S1 can be candidate SNP markers of a decrease in male reproductive potential.

**The human** ***ASMTL*** **gene** codes for acetylserotonin O-methyltransferase–like protein and carries five and 13 SNPs that can reduce and increase the ASMTL level, respectively, as detailed in Table S1. According to the PubMed keyword search, *ASMTL* overexpression is quite often seen in patients with autism [62]. Thus, 13 SNPs (e.g., rs760130208) seem to be candidate SNP markers of the autism-related loss of male reproductive potential (Table S1). On the basis of the same arguments, five candidate SNP markers (Table S1: e.g., rs1291628557) correspond to an increase in male reproductive potential owing to a decreased risk factor of autism [62].

**The human** ***DHRSX*** **gene** (dehydrogenase/reductase X-linked) contains three SNPs (e.g., rs1421651131) that can potentially increase the DHRSX level (Table S1). According to a PubMed keyword search, *DHRSX* overexpression is a typical marker of stroke in men, more often at their reproductive age as compared to this phenomenon in women [60]. Therefore, we predicted that three candidate SNP markers listed in Table S1 reduce male reproductive potential. In this table, we analogously predict three candidate SNP markers (e.g., rs1358454071) of elevated male reproductive potential due to low *DHRSX* expression and hence a weaker risk factor of stroke, as shown in Table S1.

**The human** ***PPP2R3B*** **gene** codes for phosphatase 2 regulatory subunit β and carries three SNPs (e.g., rs1162176371:c) and 15 SNPs (e.g., rs1162176371:a) listed in Table S1 that can respectively decrease and increase this enzyme’s amount as predicted here. After a PubMed keyword search, we learned about PPP2R3B deficiency as a physiological marker of spermatogenesis disruption during estradiol excess in a male’s body; this problem is caused, for example, by hormone pills containing synthetic 17α-ethynylestradiol [63]. Consequently, we predicted three candidate SNP markers of a reduction in male reproductive potential and 15 candidate SNP markers of its increase (Table S1).

In total, our Web service [41] selected 146 and 68 candidate SNP markers that can respectively enhance or reduce the TBP-binding affinity of promoters in protein-coding genes in PAR1 of the human Y chromosome (Table 1). This means that these prevalence rates of SNPs within PAR1 deviate statistically significantly from the whole-genome norm, where prevalence is fourfold greater for SNPs damaging TBP-sites as compared with the SNPs improving these sites [42, 64]. This deviation in male reproductive potential matches that in females [40], whereas the whole-genome norm corresponds to the neutral drift [14, 15] of the clinically proven SNP markers of diseases within TBP-sites [31] (Table 1).

### Pseudo-autosomal region 2 (PAR2) of the human Y chromosome

**The human** ***IL9R*** **gene** encoding interleukin 3 receptor subunit α contains two SNPs rs56317732 and rs945044791, which correspond to an increase and decrease in the IL9R level, as detailed in Table S2 (hereinafter: see Supplementary Results, Additional file 1) and shown in Fig. 3d. Due to a PubMed keyword search, we found that IL9R knockout mice are an animal model of human diseases at low risk of oral-antigen–induced anaphylaxis [65]. Within the framework of this model, we predicted candidate SNP markers (rs56317732 and rs945044791) of reduced and elevated male reproductive potential, respectively, as readers can see in Table S2.

**The human** ***SPRY3*** **gene** codes for sprouty RTK signaling antagonist 3, and has 10 SNPs (e.g., rs1180666684) increasing the SPRY3 level as predicted here. As for the output of a PubMed keyword search, Y-linked *SPRY3* overexpression elevates the male-specific risk of autism [66]. These data allow us to predict 10 candidate SNP markers of a reduction in male reproductive potential (Table S2).

**The human** ***VAMP7*** **gene** (vesicle-associated membrane protein 7, synonyms: tetanus neurotoxin-insensitive VAMP and synaptobrevin-like protein 1) has nine and four SNPs, which can cause *VAMP7* overexpression and underexpression, respectively, according to our calculations (Table S2). After a keyword search in PubMed, we found that *VAMP7* underexpression is a physiological marker of male anxiety [67] that can negatively affect family relationships and mother’s and children’s health [68]. Thus, we propose nine candidate SNPs markers (e.g., rs187456378) that can raise male reproductive potential and four SNPs (e.g., rs1295232988) as the markers that can diminish it (Table S2).

Looking through Table S2, within PAR2, we uncovered 20 and five candidate SNP markers that can raise and diminish, respectively, TBP affinity for promoters of protein-coding genes (Table 1). Again, SNPs of TBP-site damage occur fourfold less frequently than SNPs improving these sites; this ratio is a significant deviation from the genome-wide norm where the SNP-caused damage to TBP-sites is fourfold more frequent than SNP-caused improvement [42, 64] (Table 1). As readers can see, this finding is in line with a similar deviation reported for female reproductive potential [40].

### Male-specific Y-linked protein-coding genes paralogous to X-linked genes

**The human** ***ZFY*** **gene** encodes zinc finger protein Y-linked and contains two SNPs (rs1388535808 and rs996955491) increasing the ZFY level (Fig. 3e). A keyword search in PubMed produced a clinical report [69] that identified *ZFY* overexpression in spermatocytes as a physiological marker of meiotic arrest leading to azoospermia and infertility. Within applicability limitations of these clinical observations [69], we predicted two candidate SNP markers (rs1388535808 and rs996955491) of a decrease in male reproductive potential, as readers can see in Table S3.

**The human** ***AMELY*** **gene** codes for amelogenin Y-linked and has two SNPs (i.e., rs772325955 and rs34551924), which seem to reduce the AMELY amount as predicted here and shown in Table S3 (hereinafter: see Supplementary Results, Additional file 1). As for the PubMed keyword search, AMELY downregulation is a physiological marker of male-specific predisposition to suicide as discovered in a comparison between post-mortem peripheral blood samples obtained from male suicide completers and those from age-matched healthy living male volunteers as controls [70]. On this basis, we predicted two candidate SNP markers (rs772325955 and rs34551924) of low male reproductive potential (Table S3).

**The human** ***NLGN4Y*** **gene** encodes neuroligin 4 Y-linked and contains two SNPs (rs944043529 and rs755206048) increasing the expression of this gene and the only one (rs780844477) decreasing it. Concerning the PubMed keyword search, there is a clinical report [71] on *NLGN4Y* overexpression, which elevates the risk of autism spectrum disorders in boys and males. That is why we propose two candidate SNP markers (rs944043529 and rs755206048) of a decrease in male reproductive potential as well as one candidate SNP marker (rs780844477) increasing it, as presented in Table S3.

**The human** ***RPS4Y2*** **gene** encoding ribosomal protein S4 Y-linked 2 contains only one SNP (rs753818084) that decreases the expression of this gene as predicted here. After a PubMed keyword search, we learned that *RPS4Y2* underexpression is a physiological marker of male sterility [72]. This observation allows us to propose one candidate SNP marker (rs753818084) decreasing male reproductive potential (Table S3).

**The human** ***TBL1Y*** **gene** encodes transducin β like 1 Y-linked and carries two SNPs (rs893297657 and rs759428101), which both increase the expression of this gene, as calculated by us (Table S3). Our PubMed keyword search indicated that TBL1Y downregulation increases the risk of both cardiogenesis disorders and cardiac contractions in men [73]. Thus, we propose two candidate SNP markers (rs893297657 and rs759428101) of an increase in male reproductive potential (Table S3).

**The human** ***TMSB4Y*** **gene** (thymosin β4 Y-linked) carries only one SNP (rs556848823) that raises the TMSB4Y level, as shown in Table S3. Using a PubMed keyword search, we found that *TMSB4Y* overexpression generally is tumor-suppressive in men [74]. With this in mind, we propose rs556848823 as a candidate SNP marker of an increase in male reproductive potential (Table S3).

**The human** ***USP9Y*** **gene** codes for ubiquitin-specific peptidase 9 Y-linked and contains only one SNP (rs924163369) that can cause *USP9Y* overexpression according to the output of our Web service [41]. As revealed by a PubMed keyword search, this is a male-specific physiological marker of new-onset heart failure [75]. When the clinical findings [75] are applicable, we propose rs924163369 as a candidate SNP marker of a decrease in male reproductive potential (Table S3).

**The human** ***UTY*** **gene** encodes histone demethylase UTY and carries only one SNP (rs755256822) that reduces the UTY amount (Table S3). According to a PubMed keyword search, *UTY* underexpression increases the risk of developmental defects in male embryos in UTX-deficient mice as animal models of human disorders [76]. Within the limits of this animal model [76], we predicted that candidate SNP marker rs755256822 weakens male reproductive potential (Table S3).

To summarize Table S3, we detected eight and four candidate SNP markers strengthening and weakening TBP-sites of these genes, respectively (Table 1). Again, our findings about the promoters of the analyzed set of Y-linked genes significantly contradict the genome-wide norm [42, 64] (α <  0.01) and are consistent with those in females [40].

### Unique male-specific protein-coding genes on the human Y chromosome

**The human** ***CDY2A*** **gene** encodes chromodomain protein Y-linked and carries only one SNP (rs200670724) that reduces the CDY2A level as we predicted here (Fig. 3f). According to a PubMed keyword search, CDY2A downregulation physiologically causes male maturation arrest [77]. This finding allows us to propose that candidate SNP marker rs200670724 diminishes male reproductive potential (Table S4; hereinafter: see Supplementary Results, Additional file 1).

**The human** ***KDM5D*** **gene** encoding lysine demethylase 5D contains three SNPs (e.g., rs113917966) that reduce the expression of this gene (Table S4). According to a PubMed keyword search, KDM5D underexpression occurs in patients with prostate cancer often enough [78] to propose these three SNPs as candidate SNP markers of a decrease in male reproductive potential (Table S4).

**The human** ***TSPY2*** **gene** codes for testis-specific protein Y-linked 2 and has two SNPs (rs1348409621 and rs13557382090) elevating the TSPY2 amount and only one SNP (rs754865271) diminishing it (Table S4). Surprisingly, our keyword search in PubMed resulted in a clinical report [79] on both overexpression and underexpression of this protein as physiological markers of infertility in males. That is why we predicted that three candidate SNP markers (rs1348409621, rs1355738209, and rs754865271) decrease male reproductive potential (Table S4).

**Human genes** ***TSPY4*,** ***TSPY8*, and** ***TSPY10*** (testis-specific proteins Y-linked 4, 8, and 10, respectively), whereas there is only one relevant clinical report, which shows that male infertility risk grows with *TSPY4* downregulation [80] and there is nothing about either *TSPY8* or *TSPY10* within PubMed as revealed by the standard keyword search there. Because of this obvious incompleteness of data on these genes, we made further predictions about their possible effect on male reproductive potential in three steps as follows. First, using our Web service [41] we predicted that candidate SNP marker rs1275736639 increases male reproductive potential due to *TSPY4* overexpression and the negative correlation between the TSPY4 level and male infertility [80] (Table S4).

Next, we noticed that candidate SNP marker rs1275736639 predicted above (*TSPY4*) completely matches two unannotated SNPs rs1159358562 (*TSPY8*) and rs1434797814 (*TSPY10*) in terms of both nearest DNA surroundings and output of our Web service [41] in the cases of 70 bp proximal promoters containing these SNPs (Table S4). Within applicability limitations of the heuristic guesswork based on absolute matches with no other support, we assigned the same function to candidate SNP markers rs1159358562 (*TSPY8*) and rs1434797814 (*TSPY10*) as to rs1275736639 (*TSPY4*) (Table S4).

Finally, with the same limitations, among the remaining unannotated SNPs of *TSPY8*, in the same way we found two more candidate SNP markers (rs1384648018 and rs755556626) having respectively the same and opposite effects on male reproductive potential relative to those predicted for candidate SNP marker rs1159358562, as described in detail in Table S4.

As illustrated in Table S4, we uncovered six candidate SNP markers damaging TBP-sites and as many improving these sites according to the output of our Web service [41] (Table 1). Again, on the human Y chromosome, the occurrence of candidate SNP markers of male reproductive potential that improve or disrupt TBP-sites differs significantly from the genome-wide norm (Table 1) [42, 64] (α <  0.05, binomial distribution), as reported for women previously [40].

### In vitro selective validation

The primary experimental data from the in vitro analysis of the five selected candidate SNP markers of male reproductive potential—i.e., rs1452787381 (SHOX), rs1393008234 (GTPBP6), rs1402972626 (ASMT), rs1452787381 (ZFY), rs20067072 (CDY2A)—among all 261 such predictions in this work are exemplified in Fig. 2 using the case of rs1452787381 as well as in Additional file 4: Supplementary Electropherograms. Table 2 shows the experimentally measured values of the equilibrium dissociation constant (K_D_) of a TBP–DNA complex along with their standard error of the mean (SEM). All these data are the output of publicly available software GraphPad Prism 5 (URL: http://graphpad-prism.software.informer.com/5.01), the input of which was the dependences of reaction rates on ODN concentrations, as depicted in Fig. 2c and d.

**Fig. 4:**
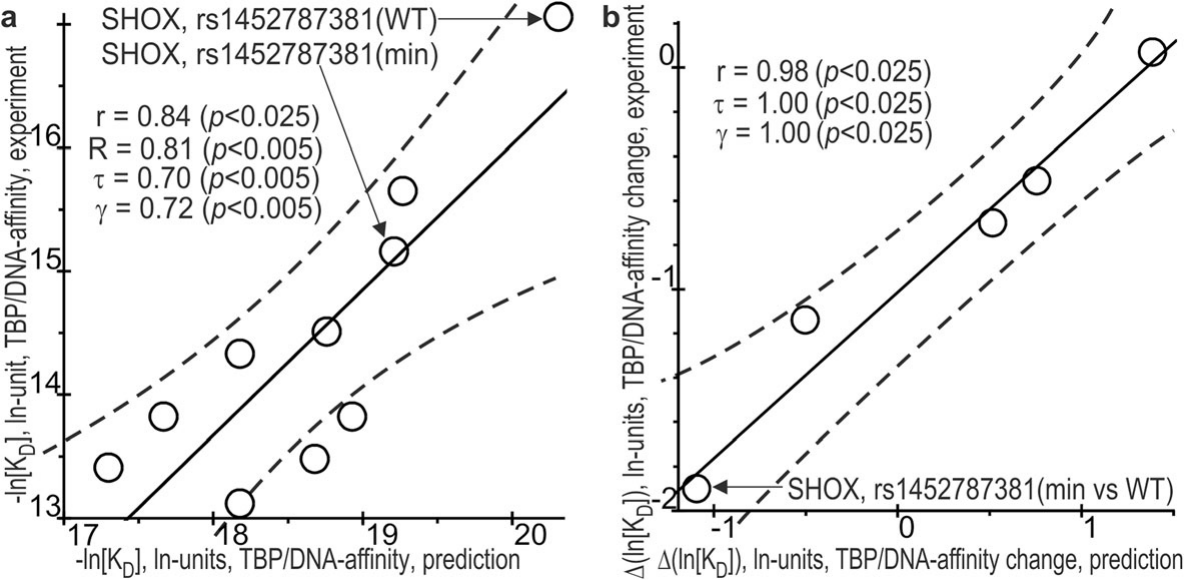
The significant correlations between the in silico predicted and in vitro experimentally measured values. **a** TBP–DNA affinity and **b** the TBP–DNA affinity change caused by the minor allele of the analyzed SNPs with respect to the norm, −ln [K_D_] and Δln [K_D_], respectively. Solid and dashed lines denote the linear regression and boundaries of its 95% confidence interval, calculated by means of software package STATISTICA (Statsoft™, Tulsa, USA); arrows pinpoint the ancestral (WT) and minor (min) alleles of the SNP being studied (rs1452787381 of *SHOX*), an analysis of which is depicted in Figs. 1 and 2 as an example of the application of our Web service SNP_TATA_Z-tester [41] in this work and its in vitro selective verification here; r, τ, γ, and *p* are coefficients of Pearson’s linear correlation, Spearman’s rank correlation, Kendall’s rank correlation, and Goodman–Kruskal generalized correlation and their *p* values, respectively

Figure 4a and b present the comparisons of our predicted (Tables S1–S4) versus experimental (Table 2) values of equilibrium dissociation constant (*K*_D_) for TBP affinity for the synthetic 26 bp ODNs identical to the human promoter regions around the the SNPs being tested, as expressed in natural logarithm units on both an absolute (i.e., −ln [K_D_]) and relative (i.e., Δln [K_D_]) scale, respectively.

As readers can see in these figures, there are statistically significant robust Pearson’s linear, Goodman–Kruskal’s generalized, Spearman’s and Kendall’s rank correlations between our computer-based predictions and experimental measurements done in this work. This proves the validity of our results on the human Y chromosome.

As a matter of discussion, the scale mismatches on the vertical (experiment) and horizontal (prediction) axes in Fig. 4 are caused by the difference in the concentrations of TBP (i.e., the uncontrolled fraction of TBP-dimers of K_D_ = 4 ± 1.5 nM [81], which do not bind DNA) here (2 nM) and in our work on optimization of the calculation model (0.3 nM) [82] used here.

### In silico validation of our genome-wide predictions as a whole

In Table 1, readers can see that the number of the candidate SNP markers damaging the TBP-sites in human Y-linked genes seems twofold less than the number of candidate SNP markers improving these sites, whereas a fourfold greater number of SNP-damaged TBP-sites than SNP-improved ones is the genome-wide norm according to ChIP-seq data analysis [42]. Many researchers have discussed countless phenomena shifting evolutionary frequencies of one type of SNPs relative to another, namely, gene conversions, coexpression and colocalization of genes, mutation initiation and fixation depending on a genomic context, and various mutagenic, pleiotropic, epistatic, and epigenetic molecular mechanisms. Effects of most of these factors drastically vary from region to region in the genome, whereas very few molecular mechanisms manifest themselves invariantly in any autosomal [40], pseudo-autosomal, and gender-specific regions (Tables 1, S1-S4), and their cumulative effect is often described using the concept of natural selection. Consequently, here we associated the deviation of the analyzed candidate SNP markers from the whole-genome norm [42, 64] with natural selection against underexpression of these genes, as shown in the middle of Table 1.

Of note, this heuristic association allows us to statistically validate our computer-based predictions as a whole, as follows. Three rightmost columns of Table 1 show the numbers of candidate SNP markers increasing (N_↑_) and decreasing (N_↓_) male reproductive potential as well as statistical significance α of their differences from one another in terms of binomial distribution. Here readers can see the statistical indistinguishability of these two diametrically opposite directions of natural selection (α >  0.06) as if male self-domestication could have happened, with its experimentally known disruptive natural selection [83]. Because there is still not enough scientific evidence that this could have happened, using Tables S1-S4 we are trying to discuss how candidate SNP markers of male reproductive potential can correspond to what is already known about pet domestication.

First, a review of comparative biology [84] associated Angelman syndrome (an autism spectrum disorder) with some differences between domesticated dogs and wild wolfs (what was denoted as “domestication syndrome”). Tables S1–S4 contain 31 candidate SNP markers of male reproductive potential depending on the risk of autism spectrum disorders in boys and men (e.g., rs1180666684).

Another review of comparative biology [85] indicates that during dog domestication, anthropogenic selection for dark colors has increased susceptibility to squamous cell carcinoma so that cancer is the leading cause of disease-related deaths among dogs, indeed. We predicted 30 candidate SNP markers that alter male reproductive potential in terms of susceptibility rates to pediatric cancer (e.g., rs779840091).

Besides, one more comparative study on wild and domestic ducks [86] suggests that the wild duck’s heart is much smaller in absolute metrics and much larger relative to body mass as compared to those in domestic ducks, whose destiny is a sedentary lifestyle until they get eaten, instead of flying from warm to cold areas in the spring and back in autumn. There are 62 candidate SNP markers of male reproductive potential that are related to cardiovascular diseases (e.g., rs944043529), which are the leading cause of death in humans [87] and are more prevalent among men versus women at reproductive age [60].

Likewise, statistical analysis of phylogenetic inertia within the hosts–infections network [88] has revealed positive correlations between the domestication time of pets and the total number of diseases that humans and pets share with each other; these data bridge the epidemiological gaps between anthropogenic worlds and wildlife. As for infections, we proposed 37 candidate SNP markers of male reproductive potential assuming that resistance to infections is a factor increasing the likelihood of raising a healthy descendant until his/her reproductive maturity (e.g., rs1419471910).

In addition, many studies on domestic dogs [89], guinea pigs [90], sheep [91], and laboratory domesticated rats [92] and mice [93] point to anxiety as one of the key behavioral traits responsible for the mutual trust within a human–pet relationship. We predicted 13 candidate SNP markers within the *VAMP7* gene that alter male reproductive potential through male-specific anxiety (e.g., rs1290051089).

Furthermore, there exists plentiful evidence of developmental changes in pets, e.g., puppy’s skull in adult dogs [94], turned-up tail and drooping ears in tame foxes [83], and robust forelimbs in domesticated horses [95]. We found nine candidate SNP markers of male reproductive potential that are related to developmental defects (e.g., rs1452787381).

Finally, after a comparison between wild boars and domestic pigs [96], we found out that spermatogenesis in pigs has become much more efficient owing to anthropogenic targeted genetic selection improving generative abilities of these meaty agricultural animals. We predicted 71 candidate SNP markers of male reproductive potential that affect spermatogenesis (e.g., rs1402972626).

To sum up, we found prototype traits of anthropogenic selection associated with animal domestication for the majority of candidate SNP markers of male reproductive potential except for eight candidate SNP markers associated with suicide (e.g., rs772325955) and verbal IQ (e.g., rs1393008234), which seem to be specific traits of humans. Overall, this finding more likely supports a self-domestication syndrome with disruptive natural selection by male reproductive potential preventing Y-linked underexpression of a protein.

## Conclusions

Here, with the help of our public Web service [31, 41], we studied all the 1206 unannotated SNPs of the proximal promoters 70 bp long in all the 63 protein-coding genes on the human Y chromosome, as shown in Table 1. We found 261 candidate SNP markers of male reproductive potential, 176 and 85 of which can respectively cause over- and underexpression of these genes. This means natural selection against underexpression of the protein products of the human Y-linked genes, as reported for female reproductive potential earlier [40]. Meanwhile, 119 and 142 among the same 261 candidate SNP markers appear to improve and diminish male reproductive potential, respectively, and these numbers are not significantly different at statistical significance α <  0.05 (for a binomial distribution), meaning statistical significance of the predictions made here as a whole. This is selection pressure in two diametrically opposite directions meeting the criteria of disruptive natural selection, which, according to Belyaev [83], is active during the domestication of animals by humans. These results allow us to conclude that during human evolution, natural selection against underexpression of the Y-linked protein-coding genes is equally increasing and decreasing male reproductive potential, as some sort of a self-domestication syndrome [84]. Currently, genome-wide research on self-domestication is a challenge that drives both genetic theory and biomedical practice [84]. For this reason, we additionally discussed the SNP-induced alterations in male reproductive potential found here versus the known differential traits seen in pets relative to their wild relatives. These phenomena showed an almost complete match, whereas verbal IQ and suicide risk are the only exceptions, which seem to be human-specific traits. Therefore, pets paired with their wild ancestors can be regarded as animal models of the diseases associated with candidate SNP markers that worsen male reproductive potential in self-domestication syndromes [84] (e.g., wild boars versus domestic pigs as an experimental model of spermatogenesis disorders in males, e.g., rs1388535808).

## Methods

### DNA sequences

We analyzed SNPs retrieved from the dbSNP database, v.151 [8] (Fig. 1b), that are within the 70 bp promoters for protein-coding transcripts from genes on the human Y chromosome. The corresponding DNA sequences are publicly available in the Ensembl database [10] in reference human genome assembly GRCh38/hg38 via Web service UCSC Genome Browser [11] (Fig. 1a).

### DNA sequence analysis in silico

Using our Web service SNP_TATA_Z-tester [41], we analyzed DNA sequences of the 70 bp promoters in front of start sites of a protein-coding transcript (where all the known TBP-sites are believed to be located [27]) of the human genes on the Y chromosome within human reference genome assembly GRCh38/hg38. For the ancestral alleles of these promoters, these data are publicly available via the Ensembl database [10] by means of the BioPerl toolkit [97] and public Web service UCSC Genome Browser [11], as shown in Fig. 1a and c: textbox “1st promoter.” For minor alleles of SNPs within the same promoters, we copied the above-mentioned wild-type DNA sequences into another textbox (2nd promoter) and then manually formatted them in accordance with database dbSNP [8] (Fig. 1). The processing of these initializing data is described in depth in Additional file 2 “Supplementary Method” [43, 82, 98–103], whereas textbox “Results” represents the outcome of our Web-service [41] (Fig. 2c).

Finally, for each significant decision on either over- or underexpression of the analyzed human genes under the influence of the SNPs being studied, we manually performed a standard keyword search in NCBI databases [45, 104] as depicted in Fig. S (see Supplementary Keyword Search, Additional file 3).

### In vitro measurements

Recombinant full-length human TBP was expressed in *Escherichia coli* BL21 (DE3) cells transformed with the pAR3038-TBP plasmid (a kind gift from Prof. B. Pugh, Pennsylvania State University) by a previously described method [105] with two modifications: the IPTG concentration was 1.0 instead of 0.1 mM; the induction time was 3 instead of 1.5 h. For details of our protocol for production and purification of human TBP, see ref. [106].

ODNs 26 bp in length were synthesized by the Biosynthesis Enterprise (Novosibirsk, Russia) and were purified by PAGE. The ODN sequences shown in Table 2 were studied here in vitro. Labeled double-stranded ODNs were prepared by ^32^P labeling of both strands by means of T4 polynucleotide kinase (SibEnzyme, Novosibirsk) with subsequent annealing by heating to 95 °C (at equimolar concentrations) and slow cooling (no less than 3 h) to room temperature. The duplexes were analyzed in a 15% nondenaturing polyacrylamide gel (1 × Tris-borate-EDTA buffer) and isolated by electroelution. For details of our protocol for labeling of ODNs with ^32^P, see ref. [106].

The equilibrium dissociation constants (K_D_) were determined for the complexes of TBP with each 26-bp ODN presented in Table 2. Experiments on association kinetics were conducted at four ODN concentrations (Fig. 2a and b as well as Additional file 4: Supplementary Electropherograms). The experiments with TBP–ODN binding were carried out at 25 °C in binding buffer (20 mM 4-[2-hydroxyethyl]-1-piperazineethanesulfonic acid [HEPES]-KOH pH 7.6, 5 mM MgCl_2_, 70 mM KCl, 1 mM dithiothreitol [DTT], 100 μg/mL BSA, 0.01% of NP-40, and 5% of glycerol) at a fixed concentration (2 nM) of active TBP. The gels were dried, and Imaging Screen-K (Kodak, Rochester, NY, USA) was exposed to these gels for analysis on a Molecular Imager PharosFX Plus phosphorimager (Bio-Rad, Herts, UK). The resulting autoradiographs were quantitated in the Quantity One 4.5.0 software (Bio-Rad) as displayed in Fig. 2c and d. Using these data as input for publicly available software Graph-Pad Prism 5 (http//graphpad-prism.software.informer.com/5.01), we calculated the equilibrium dissociation constant (K_D_). For details of our protocol for in vitro measurements of the equilibrium dissociation constant for TBP–ODN complexes, see ref. [107].

### Statistical analysis

A comparison of our predictions with the experimental values of changes in TBP–ODN affinity after the substitutions in TATA boxes was conducted by means of two options, “Multiple Regression” and “Nonparametrics,” in a standard toolbox, STATISTICA (Statsoft™, Tulsa, USA).

## Supplementary information

**Additional file 1:** Supplementary Results. **Tables S1-S4.** Candidate SNP markers of male reproductive potential within the protein-coding genes on the human Y chromosome.

**Additional file 2.** Supplementary Method. A sequence-based statistical estimate of the SNP-caused alteration in the affinity of TATA box–binding protein (TBP) for 70 bp proximal promoters of a human gene containing an SNP under study.

**Additional file 3.** Supplementary Keyword Search. Figure S. A flow chart of the keyword search for male reproductive potential components whose physiological markers correspond to alterations in the expression of human Y-linked protein-coding genes containing a given SNP under study.

**Additional file 4.** Supplementary Electropherograms. The original, raw, unfiltered, uncropped, and unedited electropherograms used for Fig. 2a and b corresponding the ancestral (left) and minor (right) alleles of the unannotated SNP rs1452787381 studied.

## Data Availability

Web service SNP_TATA_Z-tester is publicly available (URL = http://wwwmgs.bionet.nsc.ru/cgi-bin/mgs/tatascan_fox/start.pl). Within the NCBI dbSNP database build No.151 [8], which is publicly available using URL = https://www.ncbi.nlm.nih.gov/snp/, we predicted SNPs, which can reliably cause over- and underexpression of the protein-coding genes on the human Y chromosome and IDs of which are listed, as follows: rs1452787381, rs1405831103, rs1273755135, rs375938368, rs771395540, rs28378830, rs894540003, rs970127768, rs1358454071, rs1317376848, rs895063296, rs1314201179, rs1421651131, rs1448729155, rs1315266439, rs1315817680, rs977754933, rs1486365041, rs1209352981, rs1262485295, rs1420856028, rs1352067913, rs752150077, rs1288709086, rs1220344154, rs1244570562, rs1371437053, rs1276754094, rs1191037989, rs1430917370, rs1357414448, rs1285462651, rs1397856076, rs1330985228, rs192305775, rs763379654, rs1455276731, rs1473784937, rs1225019830, rs1469023312, rs1265835746, rs1485298348, rs779840091, rs1458220271, rs1390389805, rs758278463, rs1337355294, rs1207072920, rs1266314021, rs752315463, rs1172301870, rs1233753904, rs1281031474, rs746595914, rs1439781290, rs1261261445, rs757934055, rs766000936, rs1194475712, rs1463056598, rs1288116490, rs150166261, rs1359047540, rs1239446017, rs1483581212, rs1291775566, rs1435920351, rs1458842073, rs1393008234, rs1374934283, rs1330988920, rs1336077354, rs1462000578, rs1161921262, rs746504134, rs1169759938, rs772703999, rs779363374, rs1197348231, rs757522460, rs778030103, rs1206927809, rs1305502354, rs1353792558, rs756200237, rs1223931747, rs771101681, rs769069940, rs1486148098, rs1167860284, rs1455084745, rs1414365557, rs1376324319, rs1272793000, rs916987392, rs1419471910, rs1427606600, rs1240336670, rs763116366, rs1221549154, rs1265161244, rs1278813527, rs1238062584, rs1409795303, rs1414951326, rs1335638546, rs1202322215, rs1488036043, rs867349324, rs1260996736, rs4077057, rs1303845084, rs1343547775, rs766750635, rs1193086058, rs1489223460, rs1165456951, rs1207148407, rs1171696568, rs1453675169, rs1208911235, rs749731225, rs1188019448, rs1477098919, rs1420580731, rs1432958109, rs1359849378, rs777246195, rs1362480601, rs867159495, rs868524740, rs1438034084, rs894051103, rs1053955009, rs1423540571, rs1435221176, rs1465128682, rs1265767231, rs148672604, rs1484012533, rs1241748586, rs1181970017, rs1360539565, rs1234240454, rs1391922321, rs1370031793, rs1185134219, rs1423462369, rs1420856028, rs1402649633, rs1329414068, rs1402972626, rs1316071794, rs1169518250, rs749254860, rs1247910843, rs369159859, rs776937576, rs1490005750, rs769131304, rs747312680, rs1313192261, rs1280760292, rs1270130345, rs760130208, rs1219304054, rs1342636840, rs1199386338, rs1320007219, rs1271521528, rs1448375205, rs1189200229, rs1415487801, rs1156620464, rs1469404811, rs1251287274, rs1175123993, rs1291628557, rs1180366338, rs868731322, rs1271480584, rs866001797, rs1421651131, rs1448729155, rs1432712128, rs1358454071, rs867739338, rs1378563899, rs868409480, rs867299345, rs867438218, rs1419491744, rs1364333348, rs1249554398, rs1198316629, rs1462305927, rs1218528522, rs1295545779, rs1408412710, rs1182537877, rs947643665, rs1197889662, rs1039330305, rs1471195554, rs1312999970, rs1162176371, rs775448137, rs1435908201, rs56317732, rs945044791, rs1180666684, rs1253458550, rs752886077, rs1211023838, rs1301073978, rs977855071, rs1258303293, rs1486330529, rs1421114836, rs1240652420, rs187456378, rs1409364412, rs1211033675, rs1194465485, rs190225413, rs774524317, rs1290051089, rs1261057099, rs1344153396, rs1295232988, rs1303920403, rs980147704, rs1467429651, rs1388535808, rs996955491, rs772325955, rs34551924, rs944043529, rs755206048, rs780844477, rs753818084, rs893297657, rs759428101, rs556848823, rs924163369, rs755256822, rs200670724, rs113917966, rs995110746, rs1253179328, rs1348409621, rs1355738209, rs754865271, rs1275736639, rs1159358562, rs1384648018, rs755556626, and rs1434797814.

## Abbreviations

EMSA: Electrophoretic mobility shift assay
K_D_: Equilibrium dissociation constant
ln: natural logarithm
ODN: Oligodeoxyribonucleotide
PAR1 and PAR2: Pseudo-autosomal regions 1 and 2, respectively
SNP: Single-nucleotide polymorphism
TBP: TATA-binding protein
TBP-site: TBP-binding site
WT: Wild type (norm)
*AKAP17A*: A-kinase anchoring protein 17A
*AMELY*: Amelogenin Y-linked
*ASMT*: Acetylserotonin O-methyltransferase
*ASMTL*: Acetylserotonin O-methyltransferase–like
*BPY2*: *BPY2B*, and *BPY2C*, testis-specific basic charge proteins Y-linked 2, 2B, and 2C, respectively
*CD99*: CD99 molecule (synonym: Xg blood group)
*CDY1*, *CDY1B*, *CDY2A*, and *CDY2B*: Chromodomain proteins Y-linked 1, 1B, 2A, and 2B, respectively
*CRLF2*: Cytokine receptor–like factor 2
*CSF2RA*: Colony-stimulating factor 2 receptor subunit α
*DAZ1*, *DAZ2*, *DAZ3*, and *DAZ4*: deleted in azoospermia 1, 2, 3, and 4, respectively
*DDX3Y*: DEAD-box helicase 3 Y-linked
*DHRSX*: Dehydrogenase/reductase X-linked
*EIF1AY*: Eukaryotic translation initiation factor 1A Y-linked
*GTPBP6*: GTP-binding protein 6
*HSFY1* and *HSFY2*: Heat shock transcription factors Y-linked 1 and 2, respectively
*IL3RA*: Interleukin 3 receptor subunit α
*IL9R*: Interleukin 9 receptor
*KDM5D*: Lysine demethylase 5D
*NLGN4Y*: Neuroligin 4 Y-linked
*P2RY8*: P2Y receptor family member 8
*PCDH11Y*: Protocadherin 11 Y-linked
*PLCXD1*: Phosphatidylinositol-specific phospholipase C X domain–containing 1
*PPP2R3B*: Protein phosphatase 2 regulatory subunit B″β
*PRKY*: Protein kinase Y-linked (pseudogene)
*PRY*: PTPN13 like Y-linked
*PRY2*: PTPN13-like Y-linked 2
*RBMY1A1*, *RBMY1B*, *RBMY1D*, *RBMY1E*, *RBMY1F*, and *RBMY1J*: RNA-binding motif protein Y-linked family 1 members A1, B, D, E, F, and J, respectively
*RPS4Y1* and *RPS4Y2*: Ribosomal proteins S4 Y-linked 1 and 2, respectively
*SHOX*: Short stature homeobox
*SLC25A6*: Solute carrier family 25 member 6
*SPRY3*: Sprouty RTK signaling antagonist 3
*SRY*: Sex-determining region Y
*TBL1Y*: Transducin β–like 1 Y-linked
*TGIF2LY*: TGFβ-induced transcription factor 2–like protein
*TMSB4Y*: Thymosin β 4 Y-linked
*TSPY1*, *TSPY2*, *TSPY3*, *TSPY4*, *TSPY8*, *TSPY9P*, and *TSPY10*: Testis-specific proteins Y-linked 1, 2, 3, 4, 8, 9 (pseudogene), and 10, respectively
*USP9Y*: Ubiquitin-specific peptidase 9 Y-linked
*UTX* and *UTY*: Ubiquitously transcribed tetratricopeptide repeat–containing, X- and Y-linked, respectively
*VAMP7*: Vesicle-associated membrane protein 7
*VCY1B*: Variable charge Y-linked 1B
*ZBED1*: Zinc finger BED-type domain–containing protein
*ZFY*: Zinc finger protein Y-linked
