# Supplementary material for: Disruptive natural selection by male reproductive potential prevents underexpression of protein-coding genes on the human Y chromosome as a self-domestication syndrome
Source: BMC Genet. 2020 Oct 22;21(Suppl 1):89. doi: 10.1186/s12863-020-00896-6 (PMC7583315; doi:10.1186/s12863-020-00896-6)
Supplement: Supplementary file 1 — Additional file 1: Supplementary Results. Tables S1-S4. Candidate SNP markers of male reproductive potential within the protein-coding genes on the human Y chromosome. [file 12863_2020_896_MOESM1_ESM.pdf]

Additional file 1: Supplementary Results

Disruptive natural selection by male reproductive potential prevents underexpression of protein-coding genes on the human Y chromosome as a self-domestication syndrome

Mikhail Ponomarenko*, Maxim Kleshchev, Petr Ponomarenko, Irina Chadaeva, Ekaterina Sharypova, Dmitry Rasskazov, Semyon Kolmykov, Irina Drachkova, Gennady Vasiliev, Natalia Gutorova, Elena Ignatieva, Ludmila Savinkova, Anton Bogomolov, Ludmila Osadchuk, Alexandr Osadchuk, Dmitry Oshchepkov

***Correspondence:** Mikhail Ponomarenko (pon@bionet.nsc.ru)

Table S1. Candidate SNP markers of male reproductive potential in the protein genes in pseudo-autosomal region 1 (PAR1) of the human Y chromosome

Gene	dbSNP ID [8]	DNA, genome sequence				K_D, nM						hypothetical diseases	♂	[Ref]
		5′ flank	wt	m	3′ flank	**wt**	m	Δ	Z	α	ρ			
SHOX	rs1452787381	gtcgccgcgt	*a*	**g**	taaatagtga	*2*	**5**	<	17	10^{-6}	A	higher risks of disproportionate short stature and Madelung deformity	↓	[42]
	rs1405831103	gtcgccgcgt	*ataa*	**-**	atagtgagat	*2*	**8**	<	22	10^{-6}	A		↓	
	rs1273755135	tcgccgcgta	*t*	**c**	aaatagtgag	*2*	**4**	<	17	10^{-6}	A		↓	
	rs375938368	aggtcgccgc	*g*	**t**	tataaatagt	*1.5*	**1.9**	<	3	10^{-3}	B		↓	
	rs771395540	caggagccaa	*t*	**c**	aggggtcttc	*62*	**70**	<	2	0.05	D		↓	
	rs28378830	aggggtcttc	*g*	**a***	agtcaccctg	*62*	**30**	>	13	10^{-6}	A	higher risk of pathoembryo-genesis	↓	[44]
	rs894540003	caataggggt	*c*	**a**	ttcgagtcac	*62*	**23**	>	15	10^{-6}	A		↓	
	rs970127768	cgaggtcgcc	*g*	**a***	cgtataaata	*1.5*	**1.3**	>	2	0.05	D		↓	
ZBED1	rs1358454071	gcgcgcgcgt	*t*	**c**	tccgccgtcc	*126*	**209**	<	9	10^{-6}	A	in spermatozoa, at late stages of infection, higher risk of adenovirus excess re-ducing fertility	↓	[45, 46]
	rs1317376848	gccggctctt	*c*	**a**	tgcgcgcctg	*48*	**10**	>	27	10^{-6}	A	higher risk of adenovirus infection of spermatozoa, seminal, and testicular cells leading to male infertility and spontaneous abortion	↓	
	rs895063296	cgccggctct	*t*	**a**	ctgcgcgcct	*48*	**26**	>	10	10^{-6}	A		↓	
	rs1314201179	tccccgccgg	*c*	**t**	tcttctgcgc	*48*	**41**	>	3	10^{-2}	C		↓	
	rs1421651131	cgcgcgtttc	*c*	**t**	gccgtccgtc	*126*	**41**	>	20	10^{-6}	A		↓	
	rs1448729155	ggggcgcgcg	*c*	**t**	gtttccgccg	*126*	**108**	>	3	10^{-2}	C		↓	
	rs1315266439	ccattgtctg	*c*	**t**	ggcgcgggcg	*50*	**36**	>	7	10^{-6}	A		↓	
	rs1315817680	ccatgtcgcc	*a*	**t**	ttgtctgcgg	*50*	**31**	>	10	10^{-6}	A		↓	
	rs977754933	gccatgtcgc	*c*	**t**	attgtctgcg	*50*	**20**	>	19	10^{-6}	A		↓	
	rs1486365041	agccatgtcg	*c*	**t**	cattgtctgc	*50*	**37**	>	6	10^{-6}	A		↓	
	rs1209352981	gcagccatgt	*c*	**a***	gccattgtct	*50*	**18**	>	14	10^{-6}	A		↓	
	rs1262485295	GCCGGGGCAG	*c*	**t**	catgtcgcca	*50*	**27**	>	10	10^{-6}	A		↓	

Notes: hereinafter, Alleles: wt, ancestral; m, minor; "-", deletion. K_D, equilibrium dissociation constant of a TBP–DNA complex; Z, Fisher's Z-score [103]; α = 1 – p, significance (where the p value is given in Figure 1); Δ, gene expression changes: upregulation (>) and downregulation (<);ρ, heuristic rank (of candidate SNP markers) in alphabetical order from the "best" (A) to the "worst" (E). ♂, male reproductive potential: increased (↑) and reduced (↓); *)This SNP also includes other neutral alleles. Genes: *SHOX*, short stature homeobox; *ZBED1*, zinc finger BED-type–containing 1; *AKAP17A*, A-kinase anchoring protein 17A; *CSF2RA*, colony-stimulating factor 2 receptor subunit α; *CRLF2*, cytokine receptor–like factor 2; *P2RY8*, P2Y receptor family member 8; *IL3RA*, interleukin 3 receptor subunit α; *GTPBP6*, GTP-binding protein 6; *CD99*, CD99 antigen; *SLC25A6*, solute carrier family 2 member 6; *PLCXD1*, phosphatidylinositol-specific phospholipase C X domain–containing 1; ASMTs, acetylserotonin O-methyltransferase and the like; *DHRSX*, dehydrogenase/reductase X-linked; *PPP2R3B,* protein phosphatase 2 regulatory subunit β. Deletion/insertion, *DHRSX*: 9 bp = tgggcggggg; *PLCXD1*: 11bp = gccgtgttgcc; *PPP2R3B*: 10bp = cgggcgtccg; 23bp = aagcccccggggcggggcggggc; 25bp = accgaagcccccggggcggggcgg; 19bp = aagcccccggggcggggcg; 24bp = tccggggcggaccgaagcccccgg.

Table S1. Continued

Gene	dbSNP ID [8]	DNA, genome sequence				K_D, nM						hypothetical diseases	♂	[Ref]
		5′ flank	wt	m	3′ flank	**wt**	m	Δ	Z	α	ρ			
AKAP17A	rs1420856028	cggcctcttc	*c*	**t**	gcgagcccgc	*63*	**43**	>	7	10^{-6}	A	increased risk of azoospermia due to testicular degeneration	↓	[47]
	rs1352067913	aaggagaaac	*c*	**t**	cggtgctgcc	*63*	**49**	>	5	10^{-3}	B		↓	
	rs752150077	gaaggagaaa	*c*	**a,g**	ccggtgctgc	*63*	**54**	>	3	10^{-2}	C		↓	
	rs1288709086	gcaagaagga	*g*	**a**	aaacccggtg	*63*	**52**	>	3	10^{-3}	B		↓	
	rs1220344154	gggcaagaag	*g*	**a**	agaaacccgg	*63*	**39**	>	9	10^{-6}	A		↓	
	rs1244570562	agggcaagaa	*g*	**a**	gagaaacccg	*63*	**34**	>	12	10^{-6}	A		↓	
	rs1371437053	gagccagggc	*a*	**-**	agaaggagaa	*63*	**56**	>	2	0.05	D		↓	
	rs1276754094	cggagccagg	*g*	**a**	caagaaggag	*63*	**26**	>	17	10^{-6}	A		↓	
	rs1191037989	cgtcatagag	*g*	**a**	gcgggcggcg	*17*	**15**	>	2	0.05	D		↓	
	rs1430917370	gcgtcataga	*g*	**a**	ggcgggcggc	*17*	**13**	>	5	10^{-6}	A		↓	
	rs1357414448	atgcgtcata	*g*	**a**	agggcgggcg	*17*	**9**	>	12	10^{-6}	A		↓	
	rs1285462651	aagtggaaat	*g*	**a**	cgtcatagag	*17*	**9**	>	10	10^{-6}	A		↓	
	rs1397856076:**a**	tggaaatgcg	*t*	**a**	catagagggc	*17*	**13**	>	5	10^{-6}	A		↓	
	rs1397856076:**c**	tggaaatgcg	*t*	**c**	catagagggc	*17*	**22**	<	4	10^{-3}	B	reduced risk of azoospermia due to testicular degeneration in Klinefelter syndrome	↑	
	rs1330985228	aagaaggaga	*a*	**c**	acccggtgct	*63*	**75**	<	3	10^{-3}	B		↑	
	rs192305775	ggcaagaagg	*a*	**g**	gaaacccggt	*63*	**80**	<	4	10^{-3}	B		↑	
	rs763379654	tgcgtcatag	*a*	**c,g**	gggcgggcgg	*17*	**23**	<	5	10^{-3}	B		↑	
	rs1455276731	aatgcgtcat	*a*	**g**	gagggcgggc	*17*	**37**	<	13	10^{-6}	A		↑	
	rs1473784937	aaatgcgtca	*t*	**c**	agagggcggg	*17*	**37**	<	13	10^{-6}	A		↑	
P2RY8	rs1225019830	ttgctggaca	*g*	**a**	atggaactgg	*44*	**27**	>	9	10^{-6}	A	Fe excess reduces sperm quality	↓	[48, 49]
	rs1469023312	gtttcataac	*c*	**t**	gccatgcacc	*9*	**7**	>	4	10^{-3}	B		↓	
	rs1265835746	cctttgcaag	*g*	**a,c**	ttgctggaca	*44*	**56**	<	5	10^{-6}	A	lower risk of sperm quality reduced by Fe excess	↑	
	rs1485298348	cggccgcctt	*t*	**c**	gcaaggttgc	*44*	**51**	<	3	10^{-2}	C		↑	
CSF2RA	rs779840091	cagggagtgt	*a*	**g**	tgatgacaca	*10*	**23**	<	13	10^{-6}	A	reduced risk of pediatric B-cell acute lympho-blastic leukemia	↑	[50]
	rs1458220271	gagtgtatga	*t*	**c**	gacacagagg	*10*	**12**	<	2	0.05	D		↑	
	rs1390389805	agggagtgta	*t*	**c**	gatgacacag	*10*	**16**	<	7	10^{-6}	A		↑	
	rs758278463	ggggcaggga	*g*	**a**	gtatgatgac	*10*	**15**	<	4	10^{-3}	B		↑	
	rs1337355294	aggggcaggg	*a*	**g**	ggtatgatga	*10*	**12**	<	2	0.05	D		↑	
	rs1207072920	agggagctac	*t*	**g**	cagaagcggg	*23*	**37**	<	9	10^{-6}	A		↑	
	rs1266314021	gaagggagct	*a*	**g**	ctcagaagcg	*23*	**37**	<	9	10^{-6}	A		↑	
	rs752315463	actgtgtgat	*a*	**c,g**	ttttctctcc	*19*	**31**	<	10	10^{-6}	A		↑	
	rs1172301870	tcccaatcct	*a*	**g**	tgaaatgagg	*8*	**10**	<	3	10^{-2}	C		↑	
	rs1233753904	gggagctact	*c*	**t**	agaagcggga	*23*	**6**	>	20	10^{-6}	A	higher risk of pediatric B-cell acute lympho-blastic leukemia	↓	
	rs1281031474	agtgtatgat	*g*	**t**	acacagagga	*23*	**14**	>	9	10^{-6}	A		↓	
	rs746595914	tcactgtgtg	*a*	**t**	tattttctct	*19*	**14**	>	6	10^{-6}	A		↓	
	rs1439781290	ggcagggagt	*g*	**a**	tatgatgaca	*10*	**3**	>	16	10^{-6}	A		↓	
CRLF2	rs1261261445	gttcgttgta	*g*	**c**	gtccctgagg	*14*	**10**	>	5	10^{-3}	B	higher risk of childhood B-cell acute lympho-blastic leukemia	↓	[@117]
	rs757934055	tgatctgttc	*g*	**a**	ttgtaggtcc	*14*	**13**	>	2	0.05	D		↓	
	rs766000936	gtgatctgtt	*c*	**t**	gttgtaggtc	*14*	**10**	>	5	10^{-6}	A		↓	
	rs1194475712	cttgacaagt	*g*	**a**	tatctttgaa	*10*	**4**	>	13	10^{-6}	A		↓	
	rs1463056598	ctgttcgttg	*t*	**c**	aggtccctga	*14*	**39**	<	17	10^{-6}	A	reduced risk of childhood B-cell acute lympho-blastic leukemia	↑	
	rs1288116490	tctgttcgtt	*g*	**t**	taggtccctg	*14*	**18**	<	4	10^{-3}	B		↑	
	rs150166261	gatctgttcg	*t*	**c**	tgtaggtccc	*14*	**31**	<	10	10^{-6}	A		↑	
	rs1359047540	atctttgaaa	*a*	**c**	tcaactgtca	*10*	**12**	<	3	10^{-3}	B		↑	

Additional file 1: Supplementary Results

Table S1. Continued

Gene	dbSNP ID [8]	DNA, genome sequence 5′ flank	wt	m	3′ flank	K_D, nM wt	m	Δ	Z	α	ρ	hypothetical diseases	♂	[Ref]
IL3RA	rs1239446017	tcaaagaaag	*a*	**g**	gtctttcttt	*18*	**15**	>	4	10^{-3}	B	higher risk of pediatric acute myeloid leukemia	↓	[52]
	rs1483581212	ataattttca	*a*	**g**	agaaagagtc	*18*	**16**	>	2	0.05	D		↓	
	rs1291775566	aacgggaaca	*t*	**c**	gataattttc	*18*	**14**	>	5	10^{-3}	B		↓	
	rs1435920351	gggaacatga	*t*	**-**	aattttcaaa	*18*	**27**	<	8	10^{-6}	A	reduced risk of pediatric acute myeloid leukemia	↑	
	rs1458842073	gaggttataa	*a*	**g**	acagctcaat	*3*	**4**	<	7	10^{-6}	A		↑	
GTPBP6	rs1393008234	acgagcacgt	*g*	**t**	atgaggagcg	*31*	**8**	>	26	10^{-6}	A	lesser verbal IQ that can elevate reproductive potential of men	↑	[53, 54]
	rs1374934283	cacgtgatga	*g*	**a**	gagcggcctg	*31*	**19**	>	11	10^{-6}	A		↑	
	rs1330988920	agcacgtgat	*g*	**a**	aggagcggcc	*31*	**11**	>	21	10^{-6}	A		↑	
	rs1336077354	tgatgaggag	*c*	**t***	ggcctgtggg	*31*	**34**	<	2	0.05	D	higher verbal IQ that can reduce reproductive potential of men	↓	
	rs1462000578	gagcacgtga	*t*	**c**	gaggagcggc	*31*	**39**	<	6	10^{-6}	A		↓	
	rs1161921262	atcacgagca	*c*	**a**	gtgatgagga	*31*	**36**	<	4	10^{-3}	B		↓	
CD99	rs746504134	tccctgcgcg	*c*	**t#**	tctgggcgca	*171*	**78**	>	14	10^{-6}	A	typical for men rather than for women and elevates mortality in men with septic shock	↓	[55]
	rs1169759938	cgtccctgcg	*c*	**t**	gctctgggcg	*171*	**107**	>	8	10^{-6}	A		↓	
	rs772703999	cgggaccgtc	*c*	**t**	ctgcgcgctc	*171*	**122**	>	6	10^{-6}	A		↓	
	rs779363374	ctccgggacc	*g*	**a**	tccctgcgcg	*171*	**146**	>	3	10^{-2}	C		↓	
	rs1197348231	cctgcactcc	*g*	**a,t#**	ggaccgtccc	*171*	**93**	>	11	10^{-6}	A		↓	
	rs757522460	ccctgcactc	*c*	**t**	gggaccgtcc	*171*	**126**	>	5	10^{-6}	A		↓	
	rs778030103	gccctgcact	*c*	**t#**	cgggaccgtc	*171*	**83**	>	13	10^{-6}	A		↓	
	rs1206927809	ttcgcccacg	*c*	**a**	cctgcactcc	*171*	**108**	>	8	10^{-6}	A		↓	
	rs1305502354	cttcgcccac	*g*	**a**	ccctgcactc	*171*	**96**	>	10	10^{-6}	A		↓	
	rs1353792558	ccttcgccca	*c*	**t**	gccctgcact	*171*	**146**	>	3	10^{-2}	C		↓	
	rs756200237	gccgccttcg	*c*	**t**	ccacgccctg	*171*	**57**	>	19	10^{-6}	A		↓	
	rs1223931747	tgccgccttc	*g*	**a,t**	cccacgccct	*171*	**99**	>	10	10^{-6}	A		↓	
	rs771101681	tgtcctgccg	*c*	**t**	cttcgcccac	*171*	**137**	>	4	10^{-3}	B		↓	
	rs769069940	ccttcgagtc	*c*	**t**	ccgggcttcg	*76*	**64**	>	3	10^{-2}	C		↓	
	rs1486148098	ctcacccgcc	*c*	**a**	ccttcgagtc	*76*	**66**	>	3	10^{-2}	C		↓	
	rs1167860284	ggcggggcgt	*g*	**t**	taccgtactc	*20*	**12**	>	8	10^{-6}	A		↓	
	rs1455084745	cccgcccctt	*c*	**t**	tgtgcgcgcg	*37*	**16**	>	16	10^{-6}	A		↓	
	rs1414365557	gttcccgccc	*c*	**t**	ttctgtgcgc	*37*	**26**	>	7	10^{-6}	A		↓	
	rs1376324319	cgttcccgcc	*c*	**t**	cttctgtgcg	*37*	**20**	>	4	10^{-3}	B		↓	
	rs1272793000	ccccctctgt	*c*	**a**	tcctccccgg	*37*	**13**	>	17	10^{-6}	A		↓	
	rs916987392	cgccccccttc	*g*	**c**	agtccccggg	*76*	**89**	<	3	10^{-2}	C	typical for women, not for men, and reduces septic shock mortality	↑	
	rs1419471910	ggcgtgtacc	*g*	**t**	tactcccctc	*20*	**23**	<	3	10^{-2}	C		↑	
	rs1427606600	tgagaagggg	*c*	**g**	ggggcgtgta	*20*	**25**	<	3	10^{-2}	C		↑	
SLC25A6	rs1240336670	caccaaccta	*g*	**a,c**	gccgggcgcg	*41*	**33**	>	3	10^{-2}	C	spermatocyte apoptosis as spermatogenesis disorder	↓	[56, 57]
	rs763116366	ccccccccca	*c*	**a,t**	caacctaggc	*41*	**32**	>	3	10^{-3}	B		↓	
	rs1221549154	gatcccccccc	*c*	**t***	caccaaccta	*41*	**35**	>	2	0.05	D		↓	
	rs1265161244	ccagagagat	*c*	**a***	ccccccccac	*41*	**26**	>	7	10^{-6}	A		↓	
	rs1278813527	cccaccaacc	*t*	**a**	aggccgggcg	*41*	**59**	<	6	10^{-6}	A	lower risk of spermatocyte apoptosis	↑	

Additional file 1: Supplementary Results

Table S1. Continued

Gene,	dbSNP ID [8]	DNA, genome sequence				K_D, nM, prediction						hypothetical diseases	♂	[Ref.]
		5′ flank	wt	m	3′ flank	wt	m	Δ	Z	α	ρ			
PLCXD1	rs1238062584	gcaggggggag	*g*	**a**	ggaagccgtc	*186*	**147**	>	4	10^{-3}	B	reduced risks of stroke complications, whereas in the middle age, stroke is more prevalent in men vs women	↑	[58]
	rs1409795303	gcgtgcaggg	*g*	**a**	gaggggaagc	*186*	**160**	>	2	0.05	D		↑	
	rs1414951326	gggcgtgcag	*g*	**a**	gggaggggaa	*186*	**90**	>	12	10^{-6}	A		↑	
	rs1335638546	ggagcggggg	*g*	**a, t***	cgtgcagggg	*186*	**128**	>	6	10^{-6}	A		↑	
	rs1202322215	ggccgtgcag	*g*	**a**	cggagcgggg	*186*	**94**	>	11	10^{-6}	A		↑	
	rs1488036043	aggggggcc	*g*	**a, t**	tgcaggcgga	*186*	**136**	>	5	10^{-6}	A		↑	
	rs867349324	ggaggggggg	*c*	**t**	cgtgcaggcg	*186*	**133**	>	5	10^{-6}	A		↑	
	rs1260996736	gggaggggg	*g*	**t**	ccgtgcaggc	*186*	**144**	>	4	10^{-3}	B		↑	
	rs4077057	acagccaatc	*g*	**a**	cagcggactg	*68*	**51**	>	5	10^{-6}	A		↑	
	rs1303845084	cgcgccacag	*c*	**t**	caatcgcagc	*68*	**43**	>	8	10^{-6}	A		↑	
	rs1343547775	cctggtggca	*g*	**t**	gagtgtccgc	*68*	**23**	>	19	10^{-6}	A		↑	
	rs766750635	tgtccctcct	*c*	**t***	ctggtggcag	*68*	**51**	>	5	10^{-6}	A		↑	
	rs1193086058	ggcgggctgt	*c*	**a**	cctcctcctg	*68*	**35**	>	10	10^{-6}	A		↑	
	rs1489223460	attcacgtag	*c*	**t**	ccgcgaaaaa	*16*	**12**	>	4	10^{-3}	B		↑	
	rs1165456951	tgtcattcac	*g*	**a**	tagcccgcga	*16*	**12**	>	4	10^{-3}	B		↑	
	rs1207148407	gtgtgtttta	*g*	**c**	gaagagtgtc	*14*	**12**	>	3	10^{-2}	C		↑	
	rs1171696568	gactgggtgt	*g*	**a**	ttttaggaag	*14*	**7**	>	12	10^{-6}	A		↑	
	rs1453675169	cggactgggt	*g*	**a**	tgttttagga	*14*	**12**	>	2	10^{-2}	C		↑	
	rs1208911235	ccgtgcaggg	*g*	**a**	gagggggggcc	*189*	**140**	>	5	10^{-6}	A		↑	
	rs749731225	gccgtgcagg	*g*	**a***	ggagggggc	*189*	**98**	>	12	10^{-6}	A		↑	
	rs1188019448	ggccgtgcag	*g*	**t***	gggagggggg	*189*	**114**	>	9	10^{-6}	A		↑	
	rs1477098919	ggaggccgtg	*c*	**t**	agggggaggg	*189*	**31**	>	28	10^{-6}	A		↑	
	rs1420580731	ggaggggagg	*c*	**a***	cgtgcagggg	*189*	**89**	>	12	10^{-6}	A		↑	
	rs1432958109	ggggagggga	*g*	**a***	gccgtgcagg	*189*	**152**	>	4	10^{-3}	B		↑	
	rs1359849378	ccgtgcaggg	*g*	**a***	gaggggaggc	*189*	**161**	>	3	0.05	D		↑	
	rs777246195	gccgtgcagg	*g*	**a***	ggaggggagg	*189*	**109**	>	9	10^{-6}	A		↑	
	rs1362480601	gccgtgcagg	*c*	**t**	ggaggggagg	*189*	**109**	>	9	10^{-6}	A		↑	
	rs867159495	ggaggggagg	*c*	**a***	cgtgcagggg	*189*	**89**	>	12	10^{-6}	A		↑	
	rs868524740	ggggagggga	*g*	**a**	gccgtgcagg	*189*	**152**	>	4	10^{-3}	B		↑	
	rs1438034084	gggcaagagg	*g*	**a**	caacggtggg	*47*	**37**	>	4	10^{-3}	B		↑	
	rs894051103	tggtgtcagg	*c*	**t**	acgggtgggg	*47*	**32**	>	6	10^{-6}	A		↑	
	rs1053955009	ttggtgtcag	*g*	**t***	cacgggtggg	*47*	**37**	>	4	10^{-3}	B		↑	
	rs1423540571	ggcggccttg	*g*	**a**	tgtcaggcac	*47*	**27**	>	10	10^{-6}	A		↑	
	rs1435221176	gggcggcctt	*g*	**c**	gtggcaggca	*47*	**33**	>	6	10^{-6}	A		↑	
	rs1465128682:**a**	ggggcggcct	*t*	**a**	ggtggcaggc	*47*	**32**	>	6	10^{-6}	A		↑	
	rs1465128682:**g**	ggggcggcct	*t*	**g**	ggtggcaggc	*47*	**72**	<	7	10^{-6}	A	increased risks of stroke complications, whereas in the middle age, stroke is more prevalent in men vs women	↓	
	rs1265767231	tgagttcatg	*t*	**c**	agctggtgtt	*10*	**31**	<	21	10^{-6}	A		↓	
	rs148672604	tctgagttca	*t*	**a, c**	gtagctggtg	*10*	**12**	<	4	10^{-3}	B		↓	
	rs1484012533	atctgagttc	*a*	**g, c***	tgtagctggt	*10*	**18**	<	10	10^{-6}	A		↓	
	rs1241748586	gatctgagtt	*c*	**g**	atgtagctgg	*10*	**13**	<	5	10^{-6}	A		↓	
	rs1181970017	cagggaagat	*c*	**t**	tgagttcatg	*10*	**11**	<	2	0.05	D		↓	
	rs1360539565	gtggcaggag	*t*	**a, g**	gtccgcgcca	*68*	**99**	<	7	10^{-6}	A		↓	
	rs1234240454	tggtggcagg	*a*	**c**	gtgtccgcgc	*68*	**114**	<	9	10^{-6}	A		↓	
	rs1391922321	cctgtcattc	*a*	**t**	cgtagcccgc	*16*	**23**	<	6	10^{-6}	A		↓	
	rs1370031793	agcggagagt	*t*	**g**	taataggaaa	*3*	**5**	<	8	10^{-6}	A		↓	
	rs1185134219	gagcggagag	*t*	**g**	ttaataggaa	*3*	**6**	<	9	10^{-6}	A		↓	
	rs1423462369	ggagcggaga	*g*	**a**	tttaatagga	*3*	**4**	<	3	10^{-2}	B		↓	
	rs1420856028	ggtgggggcg	-	**11bp**	agggcaagag	*47*	**72**	<	7	10^{-6}	A		↓	
	rs1402649633	ggccttggtg	*t*	**c**	caggcacggg	*47*	**72**	<	7	10^{-6}	A		↓	
	rs1329414068	cggccttggt	*g*	**t**	gcaggcacgg	*47*	**55**	<	3	10^{-2}	C		↓	

Additional file 1: Supplementary Results

Table S1. Continued

Gene,	dbSNP ID [8]	DNA, genome sequence				K_D, nM, prediction						hypothetical diseases	♂	[Ref.]
		5′ flank	wt	**m**	3′ flank	wt	**m**	Δ	Z	α	ρ			
ASMT	rs1402972626	gacctttttgt	*g*	**a**	cccagaatag	*13*	**6**	>	13	10^{-6}	A	melatonin excess protects sperma-tozoa against oxidative DNA damage	↑	[59]
	rs1316071794	gctccttgaa	*g*	**c**	caagcgctcc	*47*	**37**	>	4	10^{-3}	B		↑	
	rs1169518250	gctctgtgct	*c*	**t**	cttgaagcaa	*47*	**31**	>	8	10^{-6}	A		↑	
	rs749254860	tgccagcagg	*c*	$\mathbf{t^{\#}}$	tctgtgctcc	*47*	**40**	>	3	10^{-2}	C		↑	
	rs1247910843	ggtggctctt	*c*	**a**	cccaccttgc	*47*	**26**	>	10	10^{-6}	A		↑	
	rs369159859	ttgaagcaag	*c*	$\mathbf{t^{\#}}$	gctccagagg	*44*	**35**	>	5	10^{-6}	A		↑	
	rs776937576	taggtttagt	*c*	$\mathbf{t^{\#}}$	aaatgggatt	*13*	**5**	>	18	10^{-6}	A		↑	
	rs1490005750	aataggttta	*g*	$\mathbf{c^{\#}}$	tcaaatggga	*13*	**10**	>	5	10^{-3}	B		↑	
	rs769131304	cccagaatag	*g*	$\mathbf{t^{\#}}$	tttagtcaaa	*13*	**8**	>	8	10^{-6}	A		↑	
	rs747312680	gcccagaata	*g*	**a**	gtttagtcaa	*13*	**9**	>	6	10^{-6}	A		↑	
	rs1313192261	gtgctccttg	*a*	**g**	agcaagcgct	*47*	**69**	<	7	10^{-6}	A	oxidative DNA damage in spermatozoa	↓	
	rs1280760292	atgcagacta	*t*	**g**	tttagggctg	*6*	**11**	<	9	10^{-6}	A		↓	
	rs1270130345	gtttagtcaa	*a*	**g**	tgggattgga	*13*	**15**	<	3	10^{-3}	B		↓	
ASMTL	rs760130208	tttttgcagc	*c*	**t**	gcgctgcgcg	*19*	**18**	>	2	0.05	D	increased risk of autism	↓	[60]
	rs1219304054	cgcctgtttt	*t*	**g**	tttgcagccg	*19*	**17**	>	3	10^{-2}	C		↓	
	rs1342636840	ggccgcctgt	-	**a**	ttttttgca	*19*	**16**	>	4	10^{-3}	B		↓	
	rs1199386338	gcgtggcctc	*g*	**a**	cggaggcggg	*248*	**153**	>	9	10^{-6}	A		↓	
	rs1320007219	ggcgtggcct	*c*	**t**	gcggaggcgg	*248*	**218**	>	2	0.05	D		↓	
	rs1271521528	gggggcgtgg	*c*	**t**	ctcgcggagg	*248*	**139**	>	10	10^{-6}	A		↓	
	rs1448375205	cgtggcctcg	*c*	**a, t**	ggaggcgggc	*248*	**158**	>	9	10^{-6}	A		↓	
	rs1189200229	gcgtggcctc	*g*	**a**	cggaggcggg	*248*	**152**	>	9	10^{-6}	A		↓	
	rs1415487801	ctgggcgtgg	*c*	**t**	ctcgcggagg	*248*	**149**	>	9	10^{-6}	A		↓	
	rs1156620464	actgggcgtg	*g*	**a**	cctcgcggag	*248*	**142**	>	10	10^{-6}	A		↓	
	rs1469404811	gactgggcgt	*g*	**t**	gcctcgcgga	*248*	**203**	>	4	10^{-3}	B		↓	
	rs1251287274	ctgaaggcgc	*g*	**t**	gactgggcgt	*248*	**127**	>	12	10^{-6}	A		↓	
	rs1175123993	cactgaaggc	*g*	$\mathbf{a^{\#}}$	cggactgggc	*248*	**127**	>	12	10^{-6}	A		↓	
	rs1291628557	ggccgcctgt	*t*	**c**	tttttgcag	*19*	**23**	<	4	10^{-3}	B	reduced risk of autism	↑	
	rs1180366338	gaaggcgcgg	*a*	**c, g**	ctgggcgtgg	*248*	**447**	<	10	10^{-6}	A		↑	
	rs868731322	ccatctccac	*g*	$\mathbf{c^{\#}}$	cccctcccac	*65*	**74**	<	2	0.05	D		↑	
	rs1271480584	ccccatctcc	*a*	**c**	cgcccctccc	*65*	**111**	<	10	10^{-6}	A		↑	
	rs866001797	ggacaccccc	*a*	**c**	tctccacgcc	*65*	**132**	<	12	10^{-6}	A		↑	
DHRSX	rs1421651131	cgcgcgtttc	*c*	**t**	gccgtccgtc	*126*	**41**	>	20	10^{-6}	A	typical for stroke in men, not in women, whereas in the middle age, stroke is more prevalent in men vs women	↓	[58]
	rs1448729155	ggggcgcgcg	*c*	**t**	gtttccgccg	*126*	**108**	>	3	10^{-2}	C		↓	
	rs1432712128	cgggggtcga	*g*	**c**	gtgtggccgg	*67*	**36**	>	11	10^{-6}	A		↓	
	rs1358454071	gcgcgcgcgt	*t*	**c**	tccgccgtcc	*126*	**209**	<	9	10^{-6}	A	typical for stroke in women, not in men, whereas in the middle age, stroke is rarer in women vs men	↑	
	rs867739338	ggtcgaggtg	*t*	**g**	ggccgggggt	*67*	**102**	<	8	10^{-6}	A		↑	
	rs1378563899	tgggcggggg	*9 bp*	**-**	ggccgggggt	*67*	**102**	<	8	10^{-6}	A		↑	
	rs868409480	ggggtcgagg	*t*	**g**	gtggccgggg	*67*	**102**	<	8	10^{-6}	A		↑	
	rs867299345	gcgggggtcg	*a*	**g**	ggtgtggccg	*67*	**102**	<	8	10^{-6}	A		↑	
	rs867438218	gggcgggggt	*c*	$\mathbf{t^{*}}$	gaggtgtggc	*67*	**75**	<	2	0.05	D		↑	

Additional file 1: Supplementary Results

Table S1. Continued

Gene,	dbSNP ID [8]	DNA, genome sequence				K_D, nM, prediction						hypothetical diseases	♂	[Ref.]
		5′ flank	wt	**m**	3′ flank		**m**	Δ	Z	α	ρ			
PPP2R3B	rs1419491744	tgttgatgcg	*c*	**t**	gctccagggc	*44*	**37**	>	3	10^{-2}	C	in a male's body, reduced risk of spermatogenesis disruption during estradiol excess caused by, for example, hormone pills containing synthetic 17α-ethynylestradiol	↑	[61]
	rs1364333348	gggacctgtt	*g*	**c**	atgcgcgctc	*44*	**35**	>	4	10^{-3}	B		↑	
	rs1249554398	tctcggggac	*c*	**t**	tgttgatgcg	*44*	**25**	>	10	10^{-6}	A		↑	
	rs1198316629	ctctcgggga	*c*	**a**	ctgttgatgc	*44*	**26**	>	8	10^{-6}	A		↑	
	rs1462305927	gggcggggcg	*10bp*	**-**	ggggcgccgc	*153*	**111**	>	6	10^{-6}	A		↑	
	rs1218528522	gggcggaccg	*23bp*	**-**	gcgggcgtcc	*153*	**107**	>	7	10^{-6}	A		↑	
	rs1295545779	tccggggcgg	*25bp*	**-**	ggcgcgggcg	*153*	**107**	>	7	10^{-6}	A		↑	
	rs1408412710	gggcggaccg	*19bp*	**-**	gggcgcgggc	*153*	**107**	>	7	10^{-6}	A		↑	
	rs1182537877	ccgaggtcgg	*24bp*	**-**	ggcggggcgg	*153*	**107**	>	7	10^{-6}	A		↑	
	rs947643665	cggaccgaag	*c*	**t**	cggaccgaag	*153*	**106**	>	8	10^{-6}	A		↑	
	rs1197889662	cggggcggac	*c*	**g**	gaagcccccg	*153*	**124**	>	4	10^{-3}	B		↑	
	rs1039330305	gtccggggcg	*g*	**c**	accgaagccc	*153*	**127**	>	4	10^{-3}	B		↑	
	rs1471195554	ggtccggggc	*g*	**a**	gaccgaagcc	*153*	**129**	>	3	10^{-2}	C		↑	
	rs1312999970	gaggtcggtc	*c*	**t**	ggggcggacc	*153*	**135**	>	2	0.05	D		↑	
	rs1162176371:a	acctgttgat	*g*	**a**	cgcgctccag	*44*	**14**	>	17	10^{-6}	A		↑	
	rs1162176371:c	acctgttgat	*g*	**c**	cgcgctccag	*44*	**53**	<	4	10^{-3}	B	in a male's body, increased risk of spermatogenesis disruption during estradiol excess caused by, for example, hormone pills containing synthetic 17α-ethynylestradiol	↓	
	rs775448137	cggggacctg	*t*	**c**	tgatgcgcgc	*44*	**101**	<	16	10^{-6}	A		↓	
	rs1435908201	ctcggggacc	*t*	**c**	gttgatgcgc	*44*	**51**	<	3	10^{-2}	C		↓	

Additional file 1: Supplementary Results

Table S2. Candidate SNP markers of male reproductive potential within the human Y-linked protein-coding genes in the pseudo-autosomal region 2 (PAR2)

Gene	dbSNP ID [8]	DNA, genome sequence: 5′ flank	wt	m	3′ flank	K_D, nM: **wt**	m	Δ	Z	α	ρ	hypothetical diseases	♂	[Ref]
IL9R	rs56317732	ccctgactta	*g*	**a**	aagattagtg	*12*	**8**	>	5	10^{-6}	A	increased risk of oral antigen-induced anaphylaxis	↓	[64]
	rs945044791	caactgctgc	*a*	**g**	gttatctcct	*10*	**15**	<	6	10^{-6}	A	reduced risk of oral antigen-induced anaphylaxis	↑	
SPRY3	rs1180666684	aacaacttac	*c*	**a, t**	ctgctgagct	*30*	**21**	>	7	10^{-6}	A	increased risk of autism, which is more frequent in men vs women	↓	[65]
	rs1253458550	aggtgaacaa	*c*	**t**	ttaccctgct	*30*	**17**	>	13	10^{-6}	A		↓	
	rs752886077	ggaggtgaac	*a*	**c**	acttaccctg	*30*	**18**	>	8	10^{-6}	A		↓	
	rs1211023838	gaggaggagg	*18bp*	**-**	acaacttacc	*30*	**17**	>	11	10^{-6}	A		↓	
	rs1301073978	agaaagagga	*7bp*	**-**	aacaacttac	*30*	**21**	>	7	10^{-6}	A		↓	
	rs977855071	aagaggagga	*g*	**t***	gtgaacaact	*30*	**25**	>	3	10^{-2}	C		↓	
	rs1258303293	ggaggaggag	-	**aa**	aaagaggagg	*30*	**24**	>	5	10^{-3}	B		↓	
	rs1486330529	aggaggagga	*g*	**c**	aaagaggagg	*30*	**20**	>	8	10^{-6}	A		↓	
	rs1421114836	agaggaggag	*g*	**t**	aggagaaaga	*30*	**16**	>	12	10^{-6}	A		↓	
	rs1240652420	ggaggaggag	*g*	**t**	agagagagga	*30*	**17**	>	10	10^{-6}	A		↓	
VAMP7	rs187456378	atgcggaagt	*c*	**a***	agcggcgtcc	*32*	**23**	>	5	10^{-6}	A	reduced risk of male anxiety negatively affecting family relationships and both mother's and children's health	↑	[66, 67]
	rs1409364412	gcttcatgcg	-	**cat**	gaagtcagcg	*32*	**24**	>	4	10^{-3}	B		↑	
	rs1211033675	cgcttcatgc	*g*	**a**	gaagtcagcg	*32*	**26**	>	3	10^{-2}	C		↑	
	rs1194465485	acgcgtagac	*g*	**t**	gggcgcttca	*32*	**27**	>	2	0.05	D		↑	
	rs190225413	aagaggccac	*g*	**a**	cgtagacggg	*32*	**23**	>	3	10^{-3}	B		↑	
	rs774524317	caggcctagc	*c*	**t**	gtgtcgcctg	*78*	**33**	>	17	10^{-6}	A		↑	
	rs1290051089	gcaggcctag	*c*	**a**	cgtgtcgcct	*78*	**21**	>	23	10^{-6}	A		↑	
	rs1261057099	agcaggccta	*g*	**a**	ccgtgtcgcc	*78*	**47**	>	9	10^{-6}	A		↑	
	rs1344153396	gctgggaacg	*c*	**a**	agcaggccta	*78*	**64**	>	4	10^{-3}	B		↑	
	rs1295232988	aagaggccac	*21bp*	**-**	gcggaagtca	*11*	**96**	<	6	10^{-6}	A	men anxiety negatively affecting both mother's and children's health	↓	
	rs1303920403	gcctgctgcc	*a*	**g**	ttggaggagc	*78*	**101**	<	5	10^{-6}	A		↓	
	rs980147704	ggcgcttcat	*g*	**c**	cggaagtcag	*33*	**38**	<	4	10^{-3}	B		↓	
	rs1467429651	ggggcgcttc	*a*	**c**	tgcggaagtc	*33*	**55**	<	9	10^{-6}	A		↓	

Notes: see "Notes" under Table S1; Genes: *IL9R*, interleukin 3 receptor subunit α; *SPRY3* sprouty RTK signaling antagonist 3; *VAMP7*, vesicle-associated membrane protein 7. Deletions, *SPRY3*: 18bp = agaaagaggaggaggtga; 7bp = ggaggtg; *VAMP7*: 21bp = gcgtagacggggcgcttcat.

Additional file 1: Supplementary Results

Table S3. Candidate SNP markers of male reproductive potential within the human Y-linked protein-coding genes paralogous to X-linked genes

Gene	dbSNP ID [8]	DNA, genome sequence				K_D, nM						hypothetical diseases	♂	[Ref]
		5′ flank	wt	m	3′ flank	**wt**	m	Δ	Z	α	ρ			
ZFY	rs1388535808	ggaggggcc	*c*	**t**	aactaccatc	*21*	**7**	>	15	10^{-6}	A	during removal of aberrant spermatocytes, higher risk of meiotic arrest leading to azoospermia and infertility	↓	[68]
	rs996955491	gggggcccaa	*c*	**a**	taccatcccg	*21*	**13**	>	8	10^{-6}	A		↓	
AMELY	rs772325955	tttgagaaga	*g*	**c**	atgagaaaag	*16*	**17**	<	2	0.05	D	higher risk of suicide in men	↓	[69]
	rs34551924	ggtgcacatg	*t*	**c**	ttgagaagag	*16*	**21**	<	6	10^{-6}	A		↓	
NLGN4Y	rs944043529	cagccaagag	*c*	**t**	acagtcggag	*69*	**18**	>	20	10^{-6}	A	higher risks of autism spectrum disorders in boys and males	↓	[70]
	rs755206048	tcagccaaga	*g*	**a**	cacagtcgga	*69*	**32**	>	14	10^{-6}	A		↓	
	rs780844477	tccctttgtt	*c*	**t**	ttcacctctg	*18*	**26**	<	7	10^{-6}	A	reduced risks of autism spectrum disorders in boys and males	↑	
RPS4Y2	rs753818084	acggttgcac	*c*	**g**	gtaaaaggag	*8*	**10**	<	3	0.05	D	higher risk of male sterility	↓	[71]
TBL1Y	rs893297657	ccctctgtcg	*c*	**t**	tgcagccgcc	*51*	**33**	>	8	10^{-6}	A	reduced risks of disorders of cardiogenesis and of cardiac contractions in men	↑	[72]
	rs759428101	caccgccagt	*g*	**a**	ccaatccctt	*51*	**18**	>	16	10^{-6}	A		↑	
TMSB4Y	rs556848823	aaatttctta	*g*	**a**	tgtttgctct	*14*	**10**	>	6	10^{-6}	A	improved tumor-suppressive function in men	↑	[73]
USP9Y	rs924163369	ttggagataa	*t*	**g**	tctggtggct	*12*	**8**	>	6	10^{-6}	A	increased risk of new-onset heart failure	↓	[74]
UTY	rs755256822	gtggagtaac	-	**t_{13}**	tttttttttt	*7*	**22**	<	16	10^{-6}	A	higher risks of developmental defects in UTX-deficient male embryos	↓	[75]

<u>Notes</u>: see "Notes" under Table S1; <u>Genes</u>: *ZFY*, zinc finger protein Y-linked; *AMELY*, amelogenin Y-linked; *NLGN4Y*, neuroligin 4 Y-linked; *RPS4Y2*, ribosomal protein S4 Y-linked 2; *TBL1Y*, transducin β–like 1 Y-linked; *TMSB4Y*, thymosin β4 Y-linked; *USP9Y,* ubiquitin-specific peptidase 9 Y-linked; *UTY,* ubiquitously transcribed tetratricopeptide repeat–containing. <u>Insertion</u>, UTY: t_{13} = ttttttttttttt.

Additional file 1: Supplementary Results

Table S4. Candidate SNP-markers of male reproductive potential within the male-specific protein-coding unique genes on the human Y-chromosome

Gene	dbSNP ID [13]	DNA, genome sequence: 5′ flank	wt	m	3′ flank	K_D, nM: **wt**	m	Δ	Z	α	ρ	candidate SNP markers	♂	[Ref]
CDY2A	rs200670724	atgttccata	*t*	**c**	aatcgtcata	*4*	**7**	<	7	10^{-6}	A	male maturation arrest	↓	[76]
KDM5D	rs113917966	gcgcagtggt	*c*	**t**	ccattttaaa	*7*	**14**	<	10	10^{-6}	A	increased risks of aggressive prostate cancer	↓	[77]
	rs995110746	acagcctttt	*a*	**g**	cggtccttcc	*10*	**17**	<	7	10^{-6}	A		↓	
	rs1253179328	cacagccttt	*t*	**c**	acggtccttc	*10*	**17**	<	7	10^{-6}	A		↓	
TSPY2	rs1348409621	gtcccttagg	*g*	**t**	ggcgcctgga	*69*	**36**	>	11	10^{-6}	A	higher risks of testicular maturation arrest	↓	[78]
	rs1355738209	cgcgcagtcc	*c*	**t**	ttagggggcg	*69*	**34**	>	13	10^{-6}	A		↓	
	rs754865271	gcgcagtccc	*t*	**c**	tagggggcgc	*69*	**137**	<	13	10^{-6}	A	higher risks of male infertility	↓	
TSPY4	rs1275736639	ctcccattga	*c*	**t**	tggccacgac	*47*	**30**	>	9	10^{-6}	A	reduced risks of spermatogenesis disorder	↑	[79]
TSPY8	rs1159358562	ctcccattga	*c*	**t**	tggccacgac	*47*	**30**	>	9	10^{-6}	A	reduced risks of spermatogenesis disorder (100% rs1275736639)	↑	
	rs1384648018	tcccttaggg	*g*	**a**	gcgcctggaa	*69*	**60**	>	2	0.05	D	reduced risks of spermatogenesis disorder (due to rs1159358562)	↑	
	rs755556626	gcagtccctt	*a*	**-**	gggggcgcct	*69*	**157**	<	16	10^{-6}	A	higher risks of spermatogenesis disorder (due to rs1159358562)	↓	
TSPY10	rs1434797814	ctcccattga	*c*	**t**	tggccacgac	*47*	**30**	>	9	10^{-6}	A	reduced risks of spermatogenesis disorder (100% rs1275736639)	↑	

Notes: see "Note" under Table S1; Genes: *CDYs*, chromodomains Y-linked; *KDM5D*, lysine demethylase 5D; *TSPYs,* testis specific proteins Y-linked;

Additional file 1: Supplementary Results

References

8. Sherry ST, Ward MH, Kholodov M, Baker J, Phan L, Smigielski EM, *et al*. dbSNP: the NCBI database of genetic variation. Nucleic Acids Res. 2001;29:308–11

42. Ramachandrappa S, Kulkarni A, Gandhi H, Ellis C, Hutt R, Roberts L et al. SHOX haploinsufficiency presenting with isolated short long bones in the second and third trimester. Eur J Hum Genet. 2018; 26:350-58.

44. Brosens E, de Jong EM, Barakat TS, Eussen BH, D'haene B, De Baere E et al. Structural and numerical changes of chromosome X in patients with esophageal atresia. Eur J Hum Genet. 2014; 22:1077-84.

45. Radko S, Koleva M, James KM, Jung R, Mymryk JS, Pelka P. Adenovirus E1A targets the DREF nuclear factor to regulate virus gene expression, DNA replication, and growth. J Virol. 2014; 88:13469-81.

46. Csata S, Kulcsar G. Virus-host studies in human seminal and mouse testicular cells. Acta Chir Hung. 1991; 32:83-90.

47. Winge SB, Dalgaard MD, Jensen JM, Graem N, Schierup MH, Juul A, Rajpert-De Meyts E, Almstrup K.. Transcriptome profiling of fetal Klinefelter testis tissue reveals a possible involvement of long non-coding RNAs in gonocyte maturation. Hum Mol Genet. 2018; 27:430-9

48. Costa M, Cruz E, Oliveira S, Benes V, Ivacevic T, Silva MJ et al. Lymphocyte gene expression signatures from patients and mouse models of hereditary hemochromatosis reveal a function of HFE as a negative regulator of CD8+ T-lymphocyte activation and differentiation in vivo. PLoS One. 2015; 10:e0124246.

49. Ammar O, Houas Z, Mehdi M. The association between iron, calcium, and oxidative stress in seminal plasma and sperm quality. Environ Sci Pollut Res Int. 2019; 26:14097-105.

50. Yano M, Imamura T, Asai D, Moriya-Saito A, Suenobu S, Hasegawa D et al. An overall characterization of pediatric acute lymphoblastic leukemia with CRLF2 overexpression. Genes Chromosomes Cancer. 2014;53:815-23.

51. Dou H, Chen X, Huang Y, Su Y, Lu L, Yu J et al. Prognostic significance of P2RY8-CRLF2 and CRLF2 overexpression may vary across risk subgroups of childhood B-cell acute lymphoblastic leukemia. Genes Chromosomes Cancer. 2017; 56:135-46

52. Stirewalt DL, Meshinchi S, Kopecky KJ, Fan W, Pogosova-Agadjanyan EL, Engel JH et al. Identification of genes with abnormal expression changes in acute myeloid leukemia. Genes Chromosomes Cancer. 2008; 47:8-20.

53. Vawter MP, Harvey PD, DeLisi LE. Dysregulation of X-linked gene expression in Klinefelter's syndrome and association with verbal cognition. Am J Med Genet B Neuropsychiatr Genet. 2007; 144B:728-34.

54. Lynn R. New evidence for dysgenic fertility for intelligence in the United States. Social Biology, 1999; 46:146-53.

55. Lefevre N, Corazza F, Duchateau J, Desir J, Casimir G. Sex differences in inflammatory cytokines and CD99 expression following in vitro lipopolysaccharide stimulation. Shock. 2012; 38: 37–42.

56. Guo X, Huang Y, Qi Y, Liu Z, Ma Y, Shao Y et al. Human cytomegalovirus miR-UL36-5p inhibits apoptosis via downregulation of adenine nucleotide translocator 3 in cultured cells. Arch Virol. 2015; 160:2483-90.

57. Liu J, Zhang J, Ren L, Wei J, Zhu Y, Duan J et al. Fine particulate matters induce apoptosis via the ATM/P53/CDK2 and mitochondria apoptosis pathway triggered by oxidative stress in rat and GC-2spd cell. Ecotoxicol Environ Saf. 2019;180:280-7.

58. Tian Y, Stamova B, Jickling GC, Xu H, Liu D, Ander BP et al. Y chromosome gene expression in the blood of male patients with ischemic stroke compared with male controls. Gend Med. 2012 Apr;9(2):68-75.e3.

59. Gonzalez-Arto M, Hamilton TR, Gallego M, Gaspar-Torrubia E, Aguilar D, Serrano-Blesa E et al. Evidence of melatonin synthesis in the ram reproductive tract. Andrology. 2016;4:163-71.

60. Firouzabadi SG, Kariminejad R, Vameghi R, Darvish H, Ghaedi H, Banihashemi S, et al. Copy number variants in patients with autism and additional clinical features: report of VIPR2 duplication and a novel microduplication syndrome. Mol Neurobiol. 2017;54:7019-27

61. Gao J, Zhang Y, Zhang T, Yang Y, Yuan C, Jia J, Wang Z. Responses of gonadal transcriptome and physiological analysis following exposure to 17α-ethynylestradiol in adult rare minnow Gobiocypris rarus. Ecotoxicol Environ Saf. 2017;141:209-15.

64. Osterfeld H, Ahrens R, Strait R, Finkelman FD, Renauld JC, Hogan SP. Differential roles for the IL-9/IL-9 receptor alpha-chain pathway in systemic and oral antigen-induced anaphylaxis. 2010; 125:469-476.e2.
65. Ning Z, Williams JM, Kumari R, Baranov PV, Moore T. Opposite expression patterns of Spry3 and p75NTR in cerebellar vermis suggest a male-specific mechanism of autism pathogenesis. Front Psychiatry. 2019; 10:416.
66. Danglot L, Zylbersztejn K, Petkovic M, Gauberti M, Meziane H, Combe R et al. Absence of TI-VAMP/Vamp7 leads to increased anxiety in mice. J Neurosci. 2012; 32:1962-8.
67. O'Brien AP, McNeil KA, Fletcher R, Conrad A, Wilson AJ, Jones D, Chan SW. New fathers' perinatal depression and anxiety-treatment options: an integrative review. Am J Mens Health. 2017;11:863-76.
68. Jan SZ, Jongejan A, Korver CM, van Daalen SKM, van Pelt AMM, Repping S, Hamer G. Distinct prophase arrest mechanisms in human male meiosis. Development. 2018; 145: dev160614.
69. Kimura A, Hishimoto A, Otsuka I, Okazaki S, Boku S, Horai T et al. Loss of chromosome Y in blood, but not in brain, of suicide completers. PLoS One. 2018; 13: e0190667.
70. Ross JL, Bloy L, Roberts TPL, Miller J, Xing C, Silverman LA, Zinn AR. Y chromosome gene copy number and lack of autism phenotype in a male with an isodicentric Y chromosome and absent NLGN4Y expression. Am J Med Genet B Neuropsychiatr Genet. 2019; doi: 10.1002/ajmg.b.32745.
71. Ahmadi Rastegar D, Sharifi Tabar M, Alikhani M, Parsamatin P, Sahraneshin Samani F, Sabbaghian M et al. Isoform-level gene expression profiles of human Y chromosome azoospermia factor genes and their X chromosome paralogs in the testicular tissue of non-obstructive azoospermia patients. J Proteome Res. 2015; 14:3595-605.
72. Meyfour A, Ansari H, Pahlavan S, Mirshahvaladi S, Rezaei-Tavirani M, Gourabi H et al. Y chromosome missing protein, TBL1Y, may play an important role in cardiac differentiation. J Proteome Res. 2017; 16:4391-402.
73. Wong HY, Wang GM, Croessmann S, Zabransky DJ, Chu D, Garay JP et al. TMSB4Y is a candidate tumor suppressor on the Y chromosome and is deleted in male breast cancer. Oncotarget. 2015; 6:44927-40.
74. Heidecker B, Lamirault G, Kasper EK, Wittstein IS, Champion HC, Breton E, et al. The gene expression profile of patients with new-onset heart failure reveals important gender-specific differences. Eur Heart J. 2010;31:1188-96.
75. Shpargel KB, Sengoku T, Yokoyama S, Magnuson T. UTX and UTY demonstrate histone demethylase-independent function in mouse embryonic development. PLoS Genet. 2012;8:e1002964.
76. Stahl PJ, Mielnik AN, Barbieri CE, Schlegel PN, Paduch DA. Deletion or underexpression of the Y-chromosome genes CDY2 and HSFY is associated with maturation arrest in American men with nonobstructive azoospermia. Asian J Androl. 2012; 14: 676-82
77. Komura K, Yoshikawa Y, Shimamura T, Chakraborty G, Gerke TA, Hinohara K et al. ATR inhibition controls aggressive prostate tumors deficient in Y-linked histone demethylase KDM5D. J Clin Invest. 2018; 128:2979-95.
78. Halder A, Kumar P, Jain M, Iyer VK. Copy number variations in testicular maturation arrest. Andrology. 2017; 5:460-72.
79. Gegenschatz-Schmid K, Verkauskas G, Stadler MB, Hadziselimovic F. Genes located in Y-chromosomal regions important for male fertility show altered transcript levels in cryptorchidism and respond to curative hormone treatment. Basic Clin Androl. 2019; 29:8.
103. Waardenberg AJ, Basset SD, Bouveret R, Harvey RP. CompGO: an R package for comparing and visualizing Gene Ontology enrichment differences between DNA binding experiments. BMC Bioinformatics. 2015;16:275
