# Supplementary material for: Disruptive natural selection by male reproductive potential prevents underexpression of protein-coding genes on the human Y chromosome as a self-domestication syndrome
Source: BMC Genet. 2020 Oct 22;21(Suppl 1):89. doi: 10.1186/s12863-020-00896-6 (PMC7583315; doi:10.1186/s12863-020-00896-6)
Supplement: Supplementary file 2 — Additional file 2. Supplementary Method. A sequence-based statistical estimate of the SNP-caused alteration in the affinity of TATA box–binding protein (TBP) for 70 bp proximal promoters of a human gene containing an SNP under study. [file 12863_2020_896_MOESM2_ESM.pdf]

Additional file 2: Supplementary Method

Disruptive natural selection by male reproductive potential prevents underexpression of protein-coding genes on the human Y chromosome as a self-domestication syndrome

Mikhail Ponomarenko*, Maxim Kleshchev, Petr Ponomarenko, Irina Chadaeva, Ekaterina Sharypova, Dmitry Rasskazov, Semyon Kolmykov, Irina Drachkova, Gennady Vasiliev, Natalia Gutorova, Elena Ignatieva, Ludmila Savinkova, Anton Bogomolov, Ludmila Osadchuk, Alexandr Osadchuk, Dmitry Oshchepkov

***Correspondence:** Mikhail Ponomarenko (pon@bionet.nsc.ru)

Supplementary Method

An estimate of the affinity of TATA-binding protein (TBP) for a 70 bp proximal promoter in human genes

The input data are two variants of the 70 bp proximal promoter DNA sequence of an SNP under study, namely: $S_{wt} = \{s^{wt}_{-70}\ldots s^{wt}_{i}\ldots s^{wt}_{-1}\}$ and $S_m = \{s^{m}_{-70}\ldots s^{m}_{i}\ldots s^{m}_{-1}\}$, where $s^{wt}_0 = s^{m}_0$ is the transcription start site (TSS) and $s^{\bullet}_i \in \{a, c, g, t\}$). Using the three-step molecular mechanism of the binding of TBP to the 70 bp region of a human gene promoter, which was first heuristically predicted within the framework of a linear approximation [81] and then observed experimentally [97], we estimated numerically "$-\ln[K_D(S_\bullet)]$" values expressed in natural-logarithm units (ln-units), e.g.

$$-\ln[K_D(S_\bullet)] = 10.9 - 0.2\ \{\ln[K_{SLIDE}(S_\bullet)K_{STOP}(S_\bullet)K_{BEND}(S_\bullet)]\}, \quad (1)$$

where K_D is the equilibrium dissociation constant expressed in moles per liter (M); 10.9 (ln-units) is nonspecific TBP–DNA affinity (10^{-5} M) as measured elsewhere [98]; 0.2 is a stoichiometric coefficient as shown elsewhere [81].

In Eq. 1, $-\ln[K_{STOP}(S_\bullet)]$ is an estimate of the equilibrium dissociation constant of the primary intermolecular recognition between TBP and the best possible TBP-binding site found at the intermediate step of the bioinformatics model used, namely:

$$\ln[K_{STOP}(S_\bullet)] = MAX_{-70 \le i \le -20;\ k \in \{-1; +1\}}\{\ \Sigma_{\ i-1 \le j \le i+13}\ w\{i, s^{\bullet}_{j;k}\}, \quad (2)$$

where $w\{i,s^{\bullet}_j\}$ is numerical weight of nucleotide $s^{\bullet}_j$ at the jth position of the TBP-binding site according to Bucher's position-weighted matrix, as published in ref. [99]; k indicates either direct (+1) or complement (-1) DNA chains.

In Eq. 1, $-\ln[K_{SLIDE}(S_\bullet)]$ is an estimate of the equilibrium dissociation constant of TBP sliding along B-helical DNA until it stops at the best potential TBP-binding site found at the initializing step of our computational model, namely:

$$\ln[K_{SLIDE}(S_\bullet)] = MEAN_{[\ \xi - 7;\ \xi + 19]}\ (0.8[TA] + 35.1\mu), \quad (3)$$

where ξ is the position of the best potential TBP-binding site found using Eq. 1; [TA] is the abundance of dinucleotide TA; the μ value of the minor-groove width of the DNA helix is expressed in angstroms as identified elsewhere [100]; 0.8 and 35.1 are linear regression coefficients [101].

In Eq. 1, $-\ln[K_{BEND}(S_\bullet)]$ is an estimate of the equilibrium dissociation constant of stabilization of the TBP–promoter complex by B-helical DNA of the best potential TBP-binding site found bending at a right angle as the final step of the computational model used, e.g.:

$$\ln[K_{BEND}(S_\bullet)] = MEAN_{[\ \xi - 7;\xi + 19];\ \text{both (+) and (−) DNA chains}}\ (0.9[TA, AA, TG, AG] + 2.5[TA, TC, TG] + 14.4), \quad (4)$$

where 0.9, 2.5, and 14.4 are linear regression coefficients [102].

Next, using all the possible substitutions, $s^{\bullet}_j \rightarrow \varphi$, at each position j within the 26 bp DNA region $[\ \xi - 7;\xi + 19]$ around the best potential TBP-binding site found, we estimated standard deviation $\delta\bullet$ of the $-\ln[K_D(S_\bullet)]$ estimates (Eq. 1) as

$$\delta_\bullet = \{(\Sigma_{\xi-7 \le j \le \xi}\Sigma_{\varphi \in \{a,c,g,t\}}\ln[K_D(s^{\bullet}_{\xi-7}\ldots\xi\ldots s^{\bullet}_{\xi+19}) / K_D(s^{\bullet}_{\xi-7}\ldots s_j\ldots s^{\bullet}_{\xi+19})]^2\} / (3*26)]^{1/2}. \quad (5)$$

Additional file 2: Supplementary Method

Applying Eqs. 1–5 to both ancestral S_{wt} and minor S_m of the promoter under study, we calculated $-\ln[K_D(S_{wt})] \pm \delta_{wt}$ and $-\ln(K_D(S_m)) \pm \delta_m$, respectively, and, after that, computed Fisher's Z-score [103]:

$$Z = \mathrm{abs}\{\ln[K_D(S_{wt}) / K_D(S_m))] / [\delta_{wt}^2 + \delta_m^2\}^{1/2}. \quad (6)$$

Finally, in the R software [103], we transformed this Z-score value into a p value of the probability estimate of accepting the hypothesis "H_0: $K_D(S_{wt}) \neq K_D(S_m)$" and, thus, made the final decision at this statistical significance $p > 0.95$ as follows:

IF {INEQUALITY "$-\ln[K_D(S_m)] > -\ln[K_D(S_{wt})]$" is statistically significant},

THEN {DECISION is "the minor allele of the given gene is overexpressed relative to the ancestral one"};

ELSE [IF {INEQUALITY "$-\ln[K_D(S_m)] < -\ln[K_D(S_{wt})]$" is statistically significant}, (7)

THEN {DECISION is "the minor allele of this gene is underexpressed relative to the ancestral one"},]

OTHERWISE {DECISION is "alteration of the expression of this gene is insignificant"}.

Figure 1 (see Main text) shows this decision within the "Result" text box of Web service SNP_TATA_Z-tester [41].

References

41. Sharypova E, Drachkova I, Kashina E, Rasskazov D, Ponomarenko P, Ponomarenko M et al. An experimental study of the effect of rare polymorphisms of human HBB, HBD and F9 promoter TATA boxes on the kinetics of interaction with the TATA-binding protein. Vavilovskii Zhurnal Genetiki i Selektsii. 2018;22:145-52. doi: 10.18699/vj18.342

81. Ponomarenko PM, Savinkova LK, Drachkova IA, Lysova MV, Arshinova TV, Ponomarenko MP, Kolchanov NA. A step-by-step model of TBP/TATA box binding allows predicting human hereditary diseases by single nucleotide polymorphism. Dokl Biochem Biophys. 2008;419:88-92. doi:10.1134/S1607672908020117

97. Delgadillo RF, Whittington JE, Parkhurst LK, Parkhurst LJ. The TATA-binding protein core domain in solution variably bends TATA sequences via a three-step binding mechanism. Biochemistry. 2009; 48: 1801-9. doi:10.1021/bi8018724

98. Hahn S, Buratowski S, Sharp PA, Guarente L. Yeast TATA-binding protein TFIID binds to TATA elements with both consensus and nonconsensus DNA sequences. Proc Natl Acad Sci U S A. 1989; 86:5718–22.

99. Bucher P. Weight matrix descriptions of four eukaryotic RNA polymerase II promoter elements derived from 502 unrelated promoter sequences. J Mol Biol. 1990;212:563-78

100. Karas H, Knuppel R, Schulz W, Sklenar H, Wingender E. Combining structural analysis of DNA with search routines for the detection of transcription regulatory elements. Comput Applic Biosci. 1996; 12:441-6.

101. Ponomarenko M, Ponomarenko J, Frolov A, Podkolodny N, Savinkova L, Kolchanov N, Overton G. Identification of sequence-dependent features correlating to activity of DNA sites interacting with proteins. Bioinformatics. 1999; 15:687-703.

102. Ponomarenko M, Savinkova L, Ponomarenko Y, Kel' A, Titov I, Kolchanov N. Simulation of TATA box sequences in eukaryotes. Mol Biol (Mosk). 1997;31:616-22.

103. Waardenberg AJ, Basset SD, Bouveret R, Harvey RP. CompGO: an R package for comparing and visualizing Gene Ontology enrichment differences between DNA binding experiments. BMC Bioinformatics. 2015;16:275
