# Supplementary material for: Disruptive natural selection by male reproductive potential prevents underexpression of protein-coding genes on the human Y chromosome as a self-domestication syndrome
Source: BMC Genet. 2020 Oct 22;21(Suppl 1):89. doi: 10.1186/s12863-020-00896-6 (PMC7583315; doi:10.1186/s12863-020-00896-6)
Supplement: Supplementary file 3 — Additional file 3. Supplementary Keyword Search. Figure S. A flow chart of the keyword search for male reproductive potential components whose physiological markers correspond to alterations in the expression of human Y-linked protein-coding genes containing a given SNP under study. [file 12863_2020_896_MOESM3_ESM.pdf]

Disruptive natural selection by male reproductive potential prevents underexpression of protein-coding genes on the human Y chromosome as a self-domestication syndrome

Mikhail Ponomarenko*, Maxim Kleshchev, Petr Ponomarenko, Irina Chadaeva, Ekaterina Sharypova, Dmitry Rasskazov, Semyon Kolmykov, Irina Drachkova, Gennady Vasiliev, Natalia Gutorova, Elena Ignatieva, Ludmila Savinkova, Anton Bogomolov, Ludmila Osadchuk, Alexandr Osadchuk, Dmitry Oshchepkov

***Correspondence:** Mikhail Ponomarenko (pon@bionet.nsc.ru)

Supplementary Keyword Search

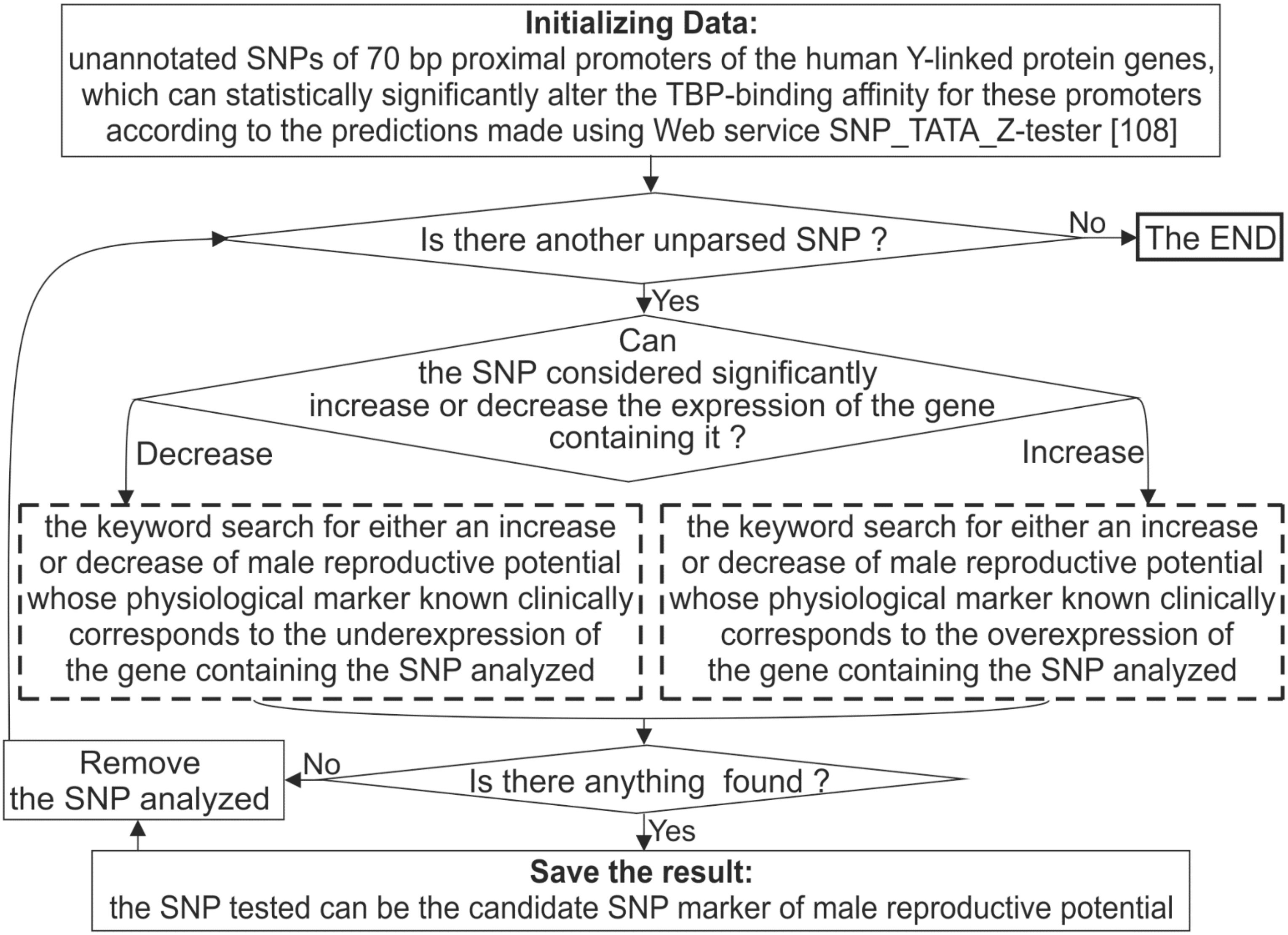

Figure S. A flow chart of the keyword search for male reproductive potential components whose physiological markers correspond to alterations in the expression of the human Y-linked protein-coding genes containing a given SNP under study.
