# Supplementary figures and images for: Disruptive natural selection by male reproductive potential prevents underexpression of protein-coding genes on the human Y chromosome as a self-domestication syndrome
Source: BMC Genet. 2020 Oct 22;21(Suppl 1):89. doi: 10.1186/s12863-020-00896-6 (PMC7583315; doi:10.1186/s12863-020-00896-6)

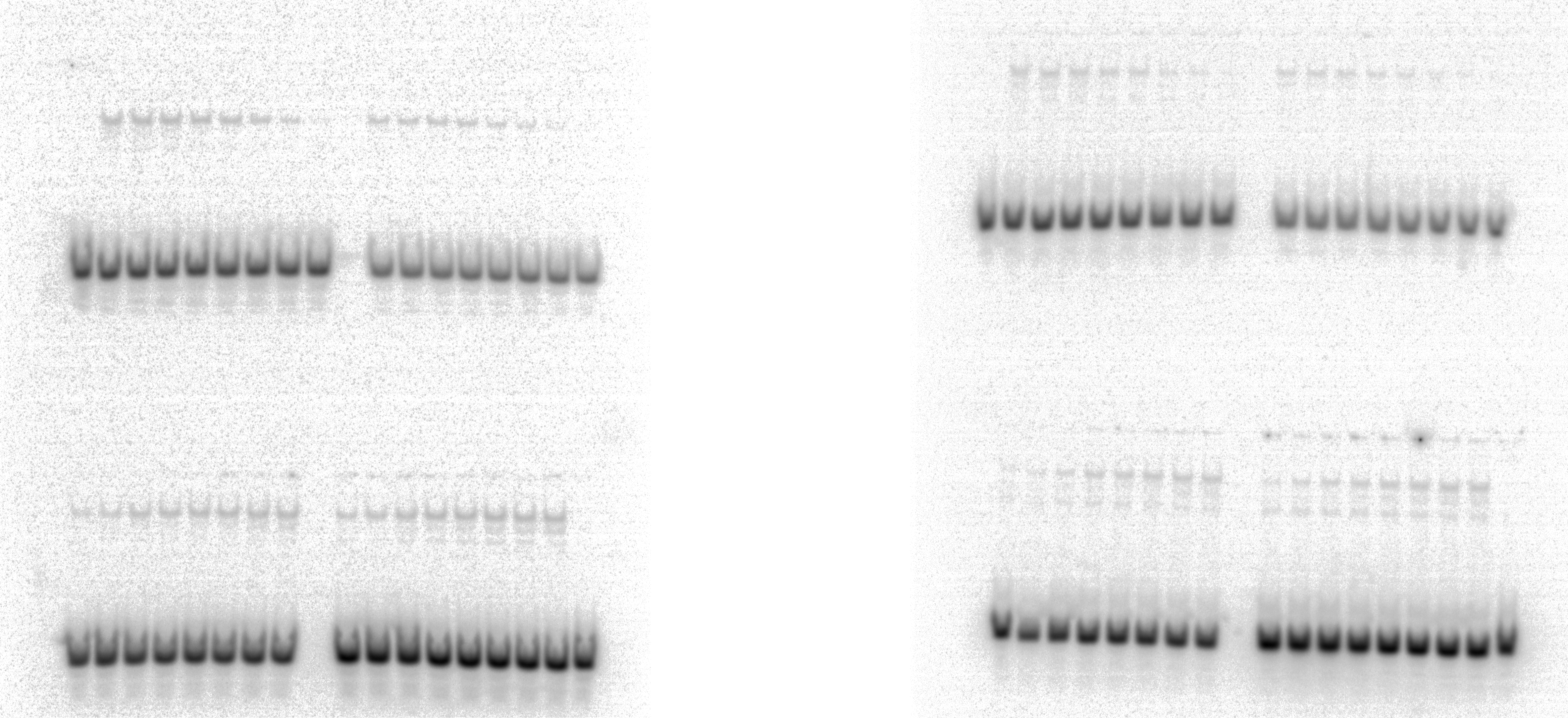

Supplement: Supplementary file 4 — Additional file 4. Supplementary Electropherograms. The original, raw, unfiltered, uncropped, and unedited electropherograms used for Fig. 2a and b corresponding the ancestral (left) and minor (right) alleles of the unannotated SNP rs1452787381 studied. [file 12863_2020_896_MOESM4_ESM.tif]
